# A comprehensive review on the potential of coumarin and related derivatives as multi-target therapeutic agents in the management of gynecological cancers

**DOI:** 10.3389/fphar.2024.1423480

**Published:** 2024-09-16

**Authors:** Gökçe Şeker Karatoprak, Berrak Dumlupınar, Engin Celep, Inci Kurt Celep, Esra Küpeli Akkol, Eduardo Sobarzo-Sánchez

**Affiliations:** ^1^ Department of Pharmacognosy, Faculty of Pharmacy, Erciyes University, Kayseri, Türkiye; ^2^ Department of Nutrition and Dietetics, Faculty of Health Sciences, Istanbul Okan University, İstanbul, Türkiye; ^3^ Department of Pharmacognosy, Faculty of Pharmacy, Acıbadem Mehmet Ali Aydinlar University, Atasehir, Istanbul, Türkiye; ^4^ Department of Biotechnology, Faculty of Pharmacy, Istanbul Okan University, Istanbul, Türkiye; ^5^ Department of Pharmacognosy, Faculty of Pharmacy, Gazi University, Ankara, Türkiye; ^6^ Instituto de Investigación y Postgrado Facultad de Ciencias de la Salud Universidad Central de Chile, Santiago, Chile; ^7^ Department of Organic Chemistry, Faculty of Pharmacy, University of Santiago de Compostela, Santiago de Compostela, Spain

**Keywords:** coumarin, drug discovery, gynecological cancer, natural compound, structure–activity relationships of coumarins

## Abstract

Current treatments for gynecological cancers include surgery, radiotherapy, and chemotherapy. However, these treatments often have significant side effects. Phytochemicals, natural compounds derived from plants, offer promising anticancer properties. Coumarins, a class of benzopyrone compounds found in various plants like tonka beans, exhibit notable antitumor effects. These compounds induce cell apoptosis, target PI3K/Akt/mTOR signaling pathways, inhibit carbonic anhydrase, and disrupt microtubules. Additionally, they inhibit tumor multidrug resistance and angiogenesis and regulate reactive oxygen species. Specific coumarin derivatives, such as auraptene, praeruptorin, osthole, and scopoletin, show anti-invasive, anti-migratory, and antiproliferative activities by arresting the cell cycle and inducing apoptosis. They also inhibit metalloproteinases-2 and -9, reducing tumor cell migration, invasion, and metastasis. These compounds can sensitize tumor cells to radiotherapy and chemotherapy. Synthetic coumarin derivatives also demonstrate potent antitumor and anticancer activities with minimal side effects. Given their diverse mechanisms of action and minimal side effects, coumarin-class phytochemicals hold significant potential as therapeutic agents in gynecological cancers, potentially improving treatment outcomes and reducing side effects. This review will aid in the synthesis and development of novel coumarin-based drugs for these cancers.

## 1 Introduction

According to epidemiological data, female genital malignancies represent a significant public health issue. While cervical, vulvar, and vaginal cancers are linked to the human papillomavirus (HPV), uterine and ovarian cancers are largely hormone-regulated cancers (Liao et al., 2019). Similar to vulvar and vaginal cancers, endometrial and ovarian cancers are mostly linked with older age, yet these latter cancer forms are still very uncommon. On the other hand, women of any age can develop cervical cancer, the most prevalent type of gynecological cancer (Patel et al., 2023). Gynecological cancer prognosis is still poor, despite the increased focus on research, prevention, and new therapeutic advances that have entered the clinical setting (D'Augè et al., 2023). Depending on the type and stage of the tumor, many first-line treatment approaches are used, the most common ones being chemotherapy and surgery. Platinum and taxane chemotherapy, antiangiogenic agents, poly (ADP-ribose) polymerase (PARP) inhibitors, tumor-intrinsic signaling pathway inhibitors, selective estrogen receptor downregulators, and immune checkpoint inhibitors are examples of novel, promising targeted agents with potential anticancer effects. These agents target the primary causes of cancer development (Woźniak et al., 2021).

Coumarins are heterocyclic compounds belonging to the benzopyrone class found in a variety of plants, including tonka bean seed (Garg et al., 2020). Furan derivatives with 4C atoms or pyran derivatives with 5C atoms are examples of oxygenated heterocyclic molecules. While pyran derivatives, which compose the structure of different chemicals, are more commonly observed, furan derivatives are rarely observed in plants. Pyran derivatives are ketonic chemicals in the form of α- or γ-pyrons. Condensation of pyron derivatives with benzene in plants produces secondary metabolites called benzo-α-pyrone (coumarin) and benzo-γ-pyrone (chromone) (Küpeli Akkol et al., 2020). It has been determined that coumarin-based compounds follow a series of complicated pathways to target a wide range of diseases by detecting their anti-Alzheimer effects with cholinesterase enzyme inhibition, antihyperglycemic effects with α-glucosidase and α-amylase enzyme inhibition, neuroprotective effects with monoamine oxidase enzyme type B (MAO-B) inhibition, and anti-inflammatory effects with cyclooxygenase and lipoxygenase enzyme inhibition. Coumarins target cancer by inhibiting different enzymes such as protein kinases, sulfatases, aromatases, caspases, and heat shock proteins. This inhibits the processes of tubulin polymerization, mitosis, and DNA replication either directly or indirectly (Singh et al., 2019). Studies showing that coumarin-derived compounds increase Ca^2+^ amounts and ROS (reactive oxygen species) generation and reduce mitochondrial membrane potential in cervical cancer have determined that coumarin causes apoptosis by reducing Bcl-xL (B-cell lymphoma-extra large) and Bcl-2 (B-cell lymphoma 2) protein levels. Activation of procaspases 3 and 9, downregulation of Bcl-2 protein, and upregulation of Bax (Bcl-2-associated X protein) protein also arresting the cell cycle at the G0/G1 phase in the ovarian cancer cell line were the identified mechanisms for a new coumarin compound, pulchrin A (Nordin et al., 2016). In research involving coumarin hybrids, coumarin-substituted benzimidazolium chlorides were used in the cell lines of ovarian cancer, while 2-imino-coumarin-based coumarin hybrids showed encouraging outcomes in ovarian and cervical cancer (Makowska et al., 2019; Karataş et al., 2019).

Approximately 80% of the approved cancer-fighting drugs are produced from natural substances. Compared to other secondary metabolites, this class of molecules is more intriguing due to its diverse chemical structures, extensive presence in nature, and the capacity to interact with a diverse set of enzymes and receptors (Song et al., 2020b). Therefore, in recent years, cancer research has focused primarily on natural and synthesized derivatives of coumarins with different structures and functional groups. Reviewing the coumarin occurrence process, its impact on gynecological cancer, structural types, and structure–activity relationship is the goal of this research.

### 1.1 Chemical classification and biosynthesis of coumarins

**FIGURE 1:**
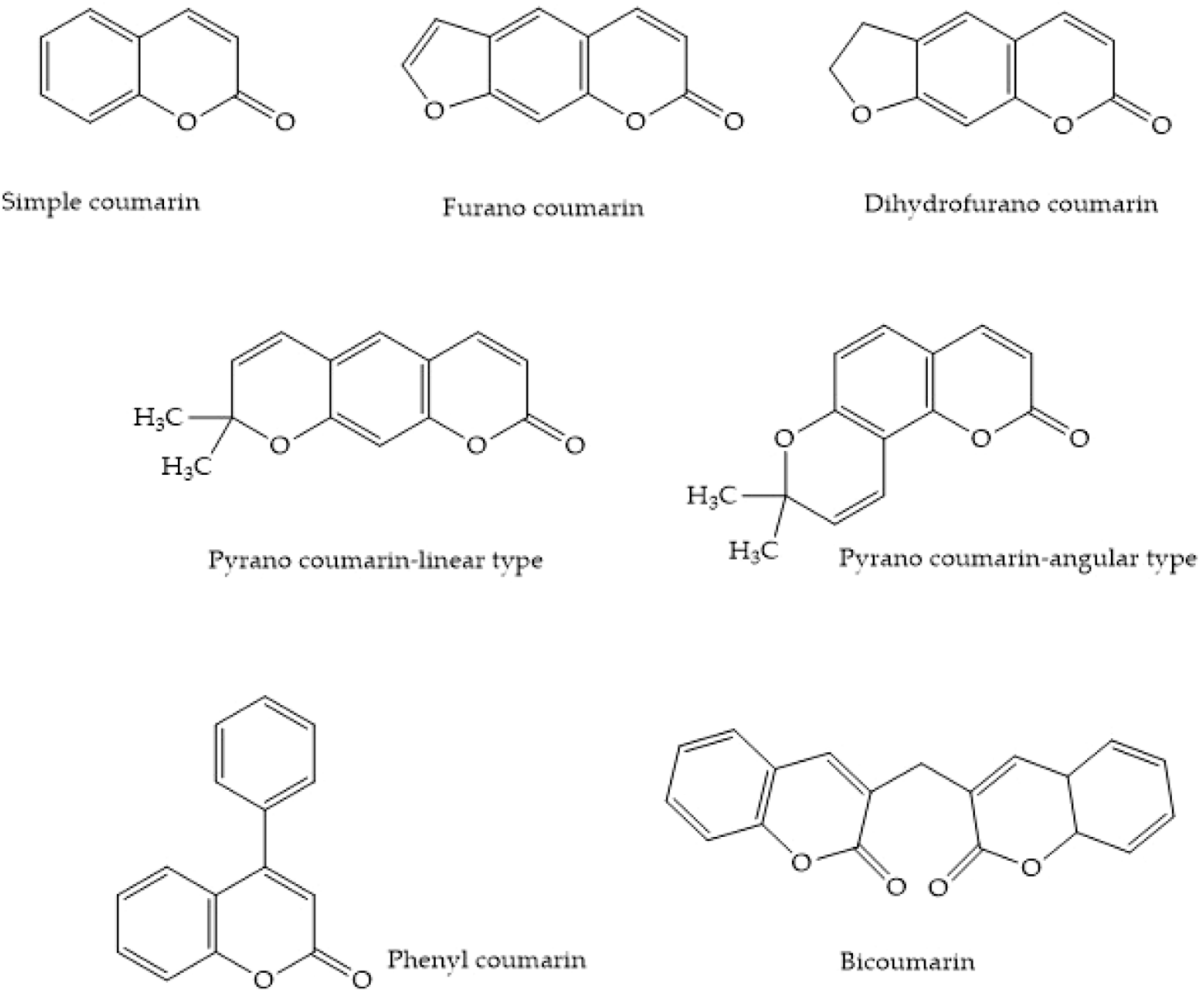
Types of coumarins.

Many plants contain coumarins (1-benzopyran-2-one), which are chemical compounds belonging to the benzopyrone class of organic chemicals. Coumarins are classified chemically into simple coumarins, furanocoumarins, pyranocoumarins, pyrone-substituted coumarins, and isocoumarins (Perez de Mello et al., 2023). The basic classification of coumarins is shown in Figure 1.

**FIGURE 2:**
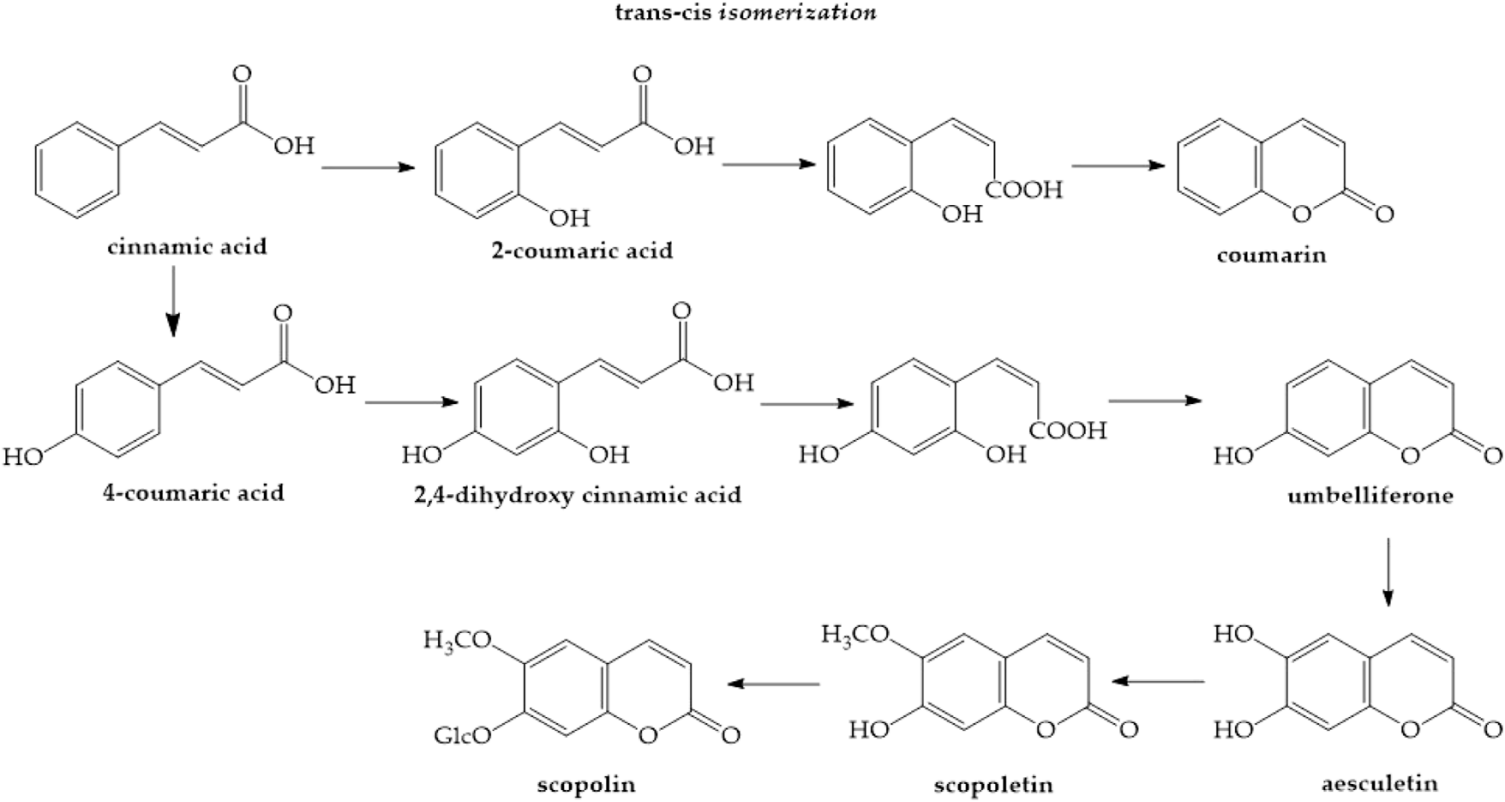
Synthesis of simple coumarins (Dewick- 2009)

The hydroxylation of cinnamic acid ortho to the side chain is an essential step in the creation of coumarins, which are cinnamic acid lactone derivatives, during biosynthesis. The aromatic ring of cinnamic acids usually undergoes direct hydroxylation. However, hydroxylation often begins with the 4-position para to the side chain and continues with consecutive hydroxylations. On the other hand, ortho to the side-chain hydroxylation of cinnamic acid or 4-coumaric acid is possible for coumarins, where the confusingly formed 2,4-dihydroxycinnamic acid appears to have the meta-hydroxylation pattern typical of phenols obtained through the acetate pathway. The side-chain structure of the two 2-hydroxycinnamic acids subsequently changes from the more stable *trans* (E) form to the less stable *cis* (Z) form. In the case of a single isolated double bond, the *trans*–*cis* isomerization would be unfavorable. However, the fully conjugated structure of cinnamic acids enables this process to occur easily. UV irradiation, such as daylight, can yield equilibrium mixtures that can be separated. Treatment with acids can lead to chemical lactonization. Coumarin biosynthesis involves enzyme-mediated *trans*–*cis* isomerization and lactonization in nature, without the need for light. Thus, the coumarins umbelliferone and coumarin are produced by cinnamic acid and 4-coumaric acid, respectively. It appears that umbelliferone is modified to produce different coumarins with additional oxygen substituents on the aromatic ring, such as esculetin and scopoletin, rather than via a common pathway from cinnamic acid to coumarin (Figure 2) (Dewick, 2009).

**FIGURE 3:**
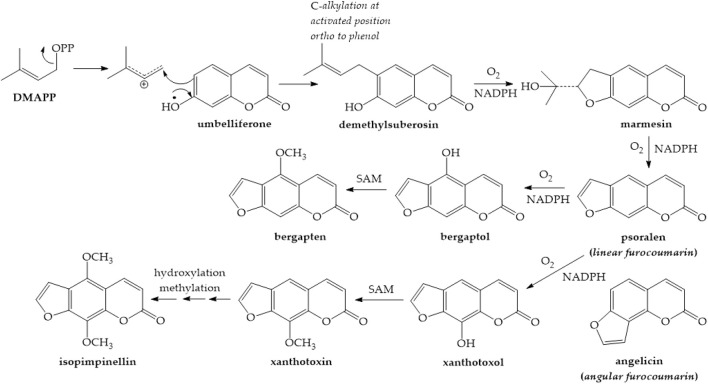
Synthesis of furanocoumarins (Dewick- 2009)

**FIGURE 4:**
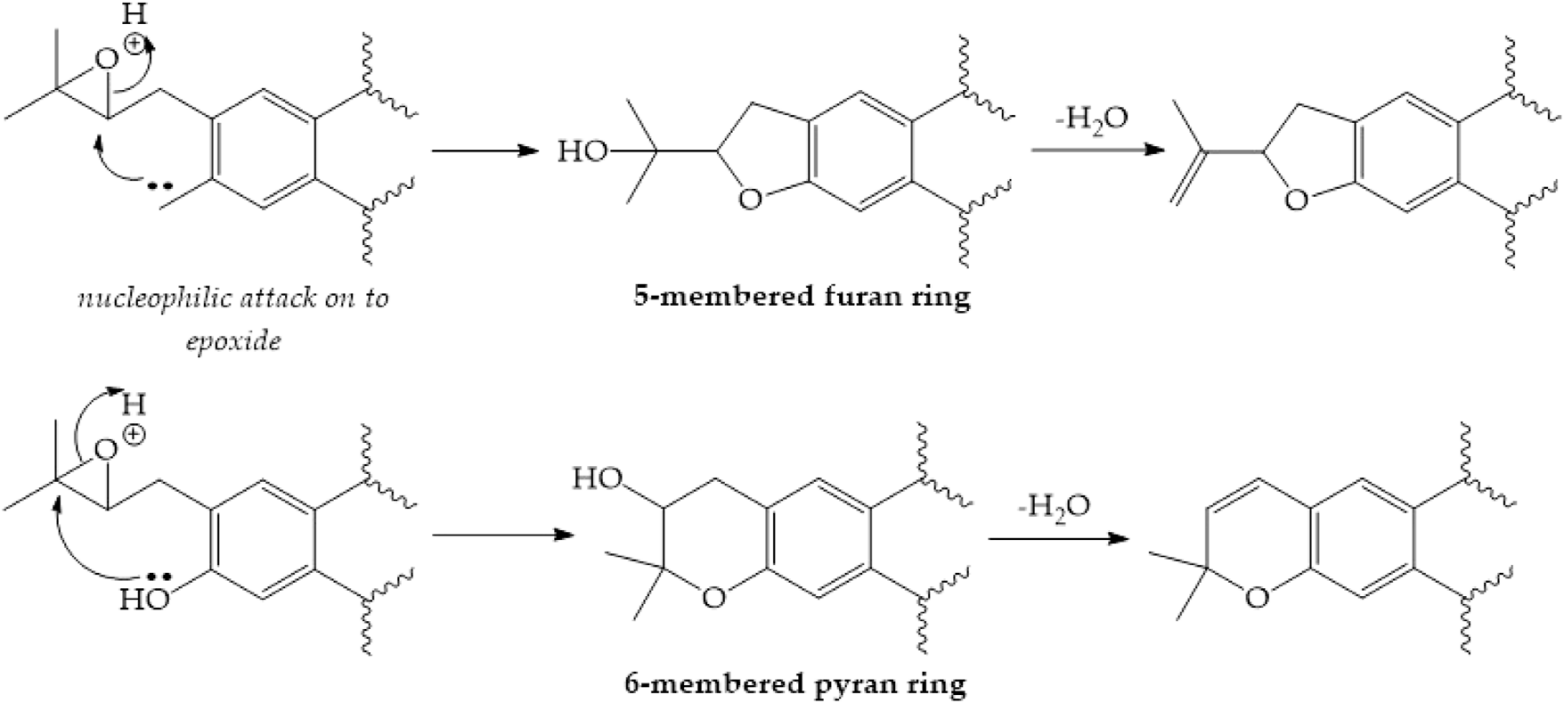
Creation of furan pyran heterocycles (Dewick- 2009)

Furanocoumarins are classified as linear psoralen and angular angelicin derivatives based on the furan ring’s alignment with the lactone structure. Umbelliferone is converted to furanocoumarins by adding a prenyl group. Umbelliferone, when subjected to dimethylallylpyrophosphate (DMAPP), the prenyl donor substrate, is converted into demethylsuberosin or osthenol by a prenyltransferase. This step is critical because it acts as the entrance point into the furanocoumarin’s biosynthetic pathway, which allows for the production of linear and angular furanocoumarins (Zou et al., 2023). The newly added DMAPP to demethylsuberosin can then cyclize with the phenol group to yield the marmesin compound. A cytochrome P-450-dependent monooxygenase catalyzes this process, and it demands NADPH and molecular oxygen as cofactors (Figure 3). For a long time, it has been proposed that cyclization involves an intermediate epoxide, with phenol’s nucleophilic attack on the epoxide group resulting in the creation of five-membered furan or six-membered pyran heterocycles often found in plants (Figure 4) (Bourgaud et al., 2006).

Pyrone-substituted coumarins are divided into three categories: 4-hydroxycoumarin (dicoumarol), 3-phenylcoumarin, and 3,4-benzocoumarin. Fermentation of sweet clover produces 4-hydroxycoumarin following the action of microorganisms on 2-coumaric acid, which reacts with formaldehyde to yield dicoumarol. The synthetic molecule warfarin is a member of the 4-hydroxycoumarin group, which is not present in plants in their free form (Dewick, 2009; Küpeli Akkol et al., 2020).

**FIGURE 5:**
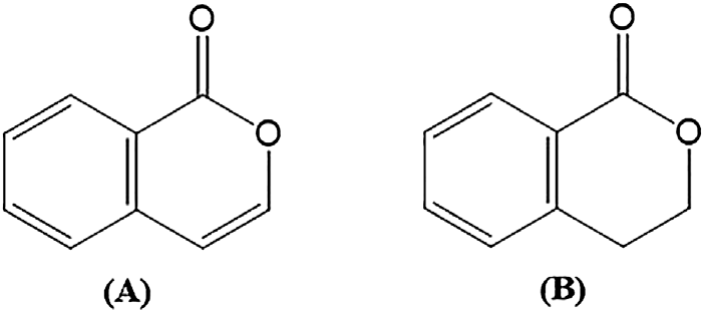
Structure of isocoumarin and 3,4-dihydroisocoumarin.

Isocoumarins (A) and 3,4-dihydroisocoumarins (B) (Figure 5) are natural lactones found in various bacteria, molds, lichens, and plants. A variety of substituted isocoumarins have been discovered in nature; however, no unsubstituted isocoumarins have been detected. Due to their characteristic process of biosynthetic origin, the majority of natural isocoumarins have a 3-alkyl (C1–C17) or a (un)substituted 3-phenyl ring on the α-pyranone ring and 8-oxygenation on the benzene ring (Saddıqa et al., 2017; Shabir et al., 2021). Chemical classification and biosynthesis of coumarins are summarized in Table 1.

**TABLE 1:** Summary of chemical classification and biosynthesis of coumarins.

Compound class	Hydroxylation/biosynthesis steps	Key enzymes/substrates	References
Coumarins (Figure 2)	Hydroxylation ortho to side chain, *trans–cis* isomerization, and lactonization	Hydroxylation ortho to side chain, *trans–cis* isomerization, and lactonization	Dewick (2009)
Furanocoumarins (Figures 3, 4)	Prenylation of umbelliferone; cyclization to marmesin	DMAPP and cytochrome P-450-dependent monooxygenase	Zou et al. (2023); Bourgaud et al. (2006)
Pyrone-substituted coumarins	Microbial action on 2-coumaric acid and reaction with formaldehyde	Formaldehyde	Dewick (2009); Küpeli Akkol et al. (2020)
Formation in bacteria, molds, lichens, and plants; 3-alkyl or substituted 3-phenyl ring; 8-oxygenation on the benzene ring (Figure 5)	Formation in bacteria, molds, lichens, and plants; 3-alkyl or substituted 3-phenyl ring; 8-oxygenation on the benzene ring	Various enzymes	Saddıqa et al. (2017); Shabir et al. (2021)

### 1.2 Overview of gynecological cancers

Gynecological cancers are among the most frequent cancers in women, making them a significant public health burden (Maheshwari et al., 2016). Gynecologic malignancies begin in a woman’s reproductive system, including the cervical, ovarian, uterine, vaginal, vulvar, and in rare cases, the fallopian tube (Ledford and Lockwood, 2019).

#### 1.2.1 Cervical cancer

Cervical cancer is the fourth most common cancer in women after breast, colorectal, and lung cancers, with approximately 90% of cases occurring in low- and middle-income countries due to a lack of vaccination and screening programs. Persistent high-risk HPV infection leads to cervical cancer development over the years (Nalbantoğlu and Arslan, 2023). The HPV genome consists of three regions: the upper regulatory region (URR), the late gene (L1 and L2), and the early gene (E1 to E7).

The persistent production of E6 and E7 proteins, which target tumor suppressor genes and CDK inhibitors to upset cell cycle regulation and cause malignant transformation, is associated with HPV’s carcinogenic potential (Motoyama et al., 2004; Christiansen et al., 2015; Boulet et al., 2007; Oyervides-Muñoz et al., 2018). L2 protein antibodies serve as antigens for distinct HPV subtypes, while the highly preserved L1 protein is used to identify HPV capsid proteins (Motoyama et al., 2004).

#### 1.2.2 Ovarian cancer

Ovarian cancer (OC) is the most devastating gynecological cancer. Despite the introduction of numerous surgical methods and treatments, the overall 5-year survival rate remains as low as 47% (Siegel et al., 2019). With several histological differences, OC has been identified as a very heterogeneous illness. The ovarian cancer-causing tumors can be broadly classified into three types: epithelial, stromal, and germ-cell tumors. The predominant histological subtypes of epithelial ovarian cancer include serous, clear cell, mucinous, and endometrioid tumors, with high-grade serous carcinomas accounting for over 68%. TP53 gene mutations are seen in roughly 80% of cases. Approximately 90% of high-grade serous carcinomas are associated with mutations in the BRCA1 (breast cancer 1) and BRCA2 (breast cancer 2) genes (Chen et al., 2003; Santin et al., 2004; Sankaranarayanan and Ferlay, 2006; Salani et al., 2008; Cho and Shih, 2009; Reid et al., 2017; Koshiyama et al., 2017; Reavis and Drapkin, 2020; Çolak et al., 2022). Clear cell carcinoma is defined by ARID1A, PIK3CA, and TERT promoter mutations, and endometrioid carcinoma is defined by PTEN and PIK3CA mutations. KRAS (Kirsten rat sarcoma virus) mutation also plays a role in mucinous carcinoma (McConechy et al., 2014).

#### 1.2.3 Uterine cancer

Uterine tumors are classified into several categories, with endometrial adenocarcinoma being the most common type due to uncontrolled estrogen exposure (Malpica et al., 2019; Onstad et al., 2016). Risk factors include obesity, early menarche, late menopause, high-calorie diets, nulliparity, diabetes, and certain genetic mutations (Braun et al., 2016). The endometrioid histotype involves WNT-β-catenin, PI3K–PTEN–AKT–mTOR, and RAS–MEK–ERK pathways and shows a high rate of microsatellite instability and POLE mutations (Bell and Ellenson, 2019).

#### 1.2.4 Vaginal cancer

Vaginal carcinomas, accounting for 2% of female genital cancers, are mostly squamous cell carcinomas (80%), followed by adenocarcinomas (15%) (Grigsby, 2002). Persistent HPV infection, especially HPV 16, is a major risk factor. E6 and E7 proteins from HPV interfere with p53 and pRB tumor suppressor proteins, promoting cancer development (Adams et al., 2021; Haręża et al., 2022). Adenocarcinomas, including clear-cell adenocarcinoma, are rare and typically affect younger women and are often linked to p53 alterations in older women (Di Donato et al., 2012; Udager et al., 2017).

#### 1.2.5 Vulvar cancer

Vulvar cancer represents 4%–5% of genital cancers. It primarily affects the labia majora. Squamous cell carcinoma is the most common type, often linked to HPV infection or chronic inflammatory conditions (Keskin and Tahta, 2021; Michalski et al., 2021). HPV-negative patients frequently show mutations in P53, CDKN2A, HRAS, and PIK3CA. Hypoxia indicators like HIF-1α and VEGF are higher in vulvar squamous cell carcinoma, suggesting their role in malignant transformation (Chokoeva et al., 2015).

#### 1.2.6 Fallopian tube cancer

Primary fallopian tube carcinoma is rare, difficult to diagnose preoperatively, and often linked to BRCA and TP53 mutations (Rexhepi et al., 2017). PARP inhibitors show efficacy in patients with BRCA mutations. The transformation begins with p53 mutations in the fallopian tube epithelium, leading to serous tubal intraepithelial carcinoma and eventual carcinoma (Stasenko et al., 2019).

### 1.3 Chemotherapy for the prevention of cancer

Chemoprevention is the use of synthetic, natural, or biological substances to stop, slow down, or even reverse the process of carcinogenesis (Woźniak et al., 2021). Chemoprevention is described by the US National Cancer Institute (NCI) as “the use of drugs, vitamins or other agents to try to reduce the risk of or delay the development or recurrence of cancer” (NCI Dictionary of Cancer Terms). In 1985, Wattenberg categorized chemopreventive substances as carcinogen formation inhibitors, blockers, and suppressors. The first group protects carcinogen synthesis from precursors, while the second group protects mutations by inhibiting particular metabolic carcinogen activation pathways and improving detoxification by neutralizing certain reactive oxygen species. The third group stops the growth and differentiation of cells and induces necrosis, autophagy, apoptosis, and other processes that impede the progression of tumors (George et al., 2021). Achieving optimum chemoprevention against gynecological cancers is still a need that requires to be satisfied for the management of this ongoing clinical issue, despite tremendous efforts. Oral contraceptives, nonsteroidal anti-inflammatory drugs (NSAIDs), PARP, retinoids, and tyrosine kinase inhibitors (TKIs) have been the most widely studied chemopreventive agents in ovarian cancer (Kathawala et al., 2018). Like breast cancer, endometrial cancer is an estrogen-induced malignancy. Progestin-containing oral contraceptive pill use has been linked to a lower incidence of endometrial cancer in the general population, as noted in observational and case–control studies (Stoffel and Walsh, 2013). The majority of clinical trials involving substances that may prevent cervical cancer have been too small, with miserable outcomes. Immune modifiers, antiviral drugs, and micronutrients are examples of possible chemopreventive medications (Sasieni, 2006).

Owing to the anticarcinogenic properties of secondary metabolites derived from natural sources that have been demonstrated in experiments, studies are being conducted to assess their potential utility in the prevention of cancer, including gynecological malignancies. Numerous secondary metabolites inhibit the growth of cancer by targeting NF-kB (nuclear factor kappa B) and AP1 (activator protein 1), blocking PKC (protein kinase C) and c-JUN NH2-terminal kinase (JNK), stopping the expression of AKT (protein kinase B), and preventing activation of PI3K (Ugbogu et al., 2013). Nonetheless, a significant obstacle for use of the majority of phytochemical chemopreventive substances is their bioavailability (Ranjan et al., 2019) Chemopreventive drugs’ metabolism may have a significant impact on their effectiveness. Apart from the well-known phase 1 and phase 2 mammalian enzymes involved in drug metabolism, it is evident that the gut microbiota has an impact on the chemopreventive drugs’ overall metabolic profile. Nanoformulations prepared with these compounds come to the fore to increase bioavailability (Dave et al., 2020).

## 2 Therapeutic approaches for gynecologic cancers and chemoresistance

Ovary, uterus, cervix, vulva, vagina, endometrium, and fallopian cancers are classified as gynecological cancers (Duarte-Franco and Franco, 2004; Zhang et al., 2023a). Early diagnosis and treatment are very important factors in the prognosis and survival of gynecological cancer. However, it is known that treatment of cancers can cause serious side effects in patients (Liu et al., 2021). In developing treatment strategies, clinical experience, the patient’s age, overall health, and nutritional performance are influential factors (Cory and Morgan, 2018). Treatment of gynecological cancer types can sometimes turn into a comorbid disease pattern (Santiago-Pérez et al., 2022). For example, while patients struggle with fear of death, serotonin levels also decrease, causing depression. In addition, cancer is a comorbid disease that causes cell toxicity in the biological process and various dysfunctions such as immune system impairment, which also causes the development of other diseases (Santiago-Pérez et al., 2022). As with other cancers, traditional and new-generation treatments are used in the treatment of gynecological cancers (Zhang et al., 2023b). Traditionally, the therapeutic approach for gynecological cancers is primarily surgical. In addition to surgery, chemotherapy, radiation therapy, and combined drug treatments are also used in gynecological cancer treatment (Kehoe, 2006; Abreu et al., 2023). Nowadays, these abovementioned treatments have been added to treatment strategies, as well as various inhibitors (Pirš et al., 2022), immunotherapies (Lorusso et al., 2021; Kobori, 2022), hormone treatments (Mitra et al., 2022) and molecular approaches (Taghizadeh et al., 2020), as summarized in Table 2.

**TABLE 2:** Summary of treatment approaches and comprehensive current practices for gynecological cancers.

Treatment strategies	Recommended applications	Side effects	References
Surgery	• Hysterectomy • Lymph node surgery • Minimal invasive surgery	• Pain • Swelling • Problem in the excretory system	• Gallotta et al. (2023) • National Comprehensive Cancer Network, (2024) ^®^ (NCCN) Guidelines Version 2.2024
Chemotherapy	• Paclitaxel • Carboplatin • Combined treatment of paclitaxel and carboplatin • Combined treatment of carboplatin and doxorubicin • Combined treatment of docetaxel and carboplatin • Combined treatment of docetaxel + oxaliplatin + bevacizumab • Combined treatment of docetaxel + carboplatin or oxaliplatin + bevacizumab	• Appetite problems • Nausea or vomiting • Hair loss • Diarrhea • Low blood cell counts • Infection • Problems of bleeding and bruising • Fevers	• Ngu et al. (2022) • National Comprehensive Cancer Network, (2024) ^®^ (NCCN) Guidelines Version 2.2024 • Ferrero et al. (2022)
Maintenance and target therapy	• Poly ADP-ribose polymerase (PARP) inhibitors for homologous recombination deficiency (HRD)-positive GCs and GCs with BRCA mutations • Olaparib (Lynparza) • Niraparib (Zejula) • Rucaparib (Rubraca) • Veliparib	• Neutropenia • Infection • Increased liver enzymes synthesis • Vomiting and nausea	• National Comprehensive Cancer Network, (2024) ^®^ (NCCN) Guidelines Version 2.2024 • Saint-Ghislain et al. (2021) • O’Malley et al. (2023)
Tyrosine kinase inhibitors (TKI)	• Apatinib • Tucatinib • Neratinib	• Cardiac toxicity • Respiratory disorders • Gastrointestinal problems • Neutropenia • Anemia • Increased liver enzymes	• Saint-Ghislain et al. (2022) • Kathawala et al. (2018)
Endocrine therapy	• Tamoxifen • Anastrozole • Exemestane • Letrozole • Leuprolide acetate • Megestrol acetate	• Pain and vaginal dryness • Insomnia • Night sweats • Hot flush • Depression	• Kobori (2022) • Matsuoka et al. (2022) • National Comprehensive Cancer Network, (2024) ^®^ (NCCN) Guidelines Version 2.2024
Biomarker-based and immunotherapy	• Combined treatment of larotrectinib (Vitrakvi) and entrectinib (Rozlytrek) for NTRK gene fusion-positive • Combined treatment of dabrafenib (Tafinlar) and trametinib (Mekinist) for BRAF V600E mutation • Pembrolizumab (Keytruda) and dostarlimab-gxly (Jemperli) for cell cycle check points • Bevacizumab, Zaltrap or ramucirumab (Cyramza) for VEGF inhibitors	• Colitis • Rash • Hypophysitis	• National Comprehensive Cancer Network, (2024) ^®^ (NCCN) Guidelines Version 2.2024 • Cardeña-Gutiérrez et al. (2022)

In gynecological cancers, surgery is usually determined based on the region and organ in which the lesions develop. The standard procedures in gynecological surgeries are staging surgery, debulking surgery, total hysterectomy, radical hysterectomy, unilateral salpingo-oophorectomy, bilateral salpingo-oophorectomy, omentectomy, lymph node removal, and minimal invasive surgery (Schlaerth and Abu-Rustum, 2006; Singh et al., 2019; Nezhat et al., 2010; Ngu et al., 2022; Cianci et al., 2022; Gallotta et al., 2023).

Chemotherapy is a drug-based therapy that stops the division and proliferation of cancer cells and causes cell death. Chemotherapy can be performed in three different ways, depending on the size and location of cancer cells: intravenous, intraperitoneal, and oral use (Reed and Sadozye, 2016; Kurnit et al., 2021; Ngu et al., 2022). In many cases of gynecological cancers, after surgery, patients are treated with platinum, anthracyclines, and similar chemotherapeutic drugs (Ngu et al., 2022; Ferrero et al., 2022). These drugs belong to a systemic drug group, and in most cases of gynecologic cancers, they are administered alone or in combination. Most of these drugs are cytotoxic and have side effects, such as killing both cancer cells and healthy cells (Rahaman et al., 2009). Platinum is composed of cisplatin and carboplatin. When carboplatin is administered to patients, the free carboplatin plasma concentration (AUC) and glomerular filtration rate (GFR) are calculated for the determination of dose (Calvert et al., 2023). Carboplatin is less nephrotoxic, neurotoxic, and emetogenic than cisplatin and is a second-generation platinum compound, as effective as cisplatin. Platinum compounds target DNA and prevent the proliferation of cancer cells as a cause of structural damage to DNA (Boulikas and Vougiouka, 2003). The platinum compounds contain CI groups, which inhibit the growth of cancer cells by inhibiting the replication, transcription, and nuclear functions of DNA, in a hydrolyzed reaction with glutathione in the cytoplasm and DNA in the nucleus (Boulikas and Vougiouka, 2003). This activates apoptosis and prevents the survival of cancer cells. Platinum compound medicines are usually administered to patients every 3 weeks as a combination with taxale group medicines. While platinum and paclitaxel are initial treatments for many other gynecological cancers, mainly ovarian cancer, chemotherapy agents have serious side effects in patients (Ngu et al., 2022). The most common side effects include neutropenia, thrombocytopenia, anemia, gastrointestinal toxicity, alopecia, neuropathy, and kidney and liver toxicities (Boulikas and Vougiouka, 2003; Rahaman et al., 2009; Reed and Sadozye, 2016; Ngu et al., 2022; Ferrero et al., 2022; Calvert et al., 2023). The general health status of patients undergoing chemotherapy should be monitored at certain times. Usually, these routine checks involve full blood tests and kidney and liver function tests, and in an unusual situation, the clinic should intervene immediately. Depending on the toxicity and the purpose of treatment, a dose reduction or a change in the chemotherapy regimen may be necessary (Chambers et al., 2021). Although clinical evidence often indicates that the paclitaxel/carboplatin combination does not cause serious neutropenic complications, long-term use may require a granulocyte colony-stimulating factor (Ngu et al., 2022). Because of the cell toxicity caused by chemotherapy, multidisciplinary applications are needed in the treatment of gynecological cancers, and another approach is radiotherapy.

The purpose of radiotherapy is based on the area where the lesions are found and the exposure of those areas to a specific wavelength of light against the potential for spread/invasion (Ngu et al., 2022). In radiotherapy, the correct planning of the dose of radiation plays an important role in achieving an effective outcome. Two types of radiation therapy are commonly used in adjuvant or post-surgical treatment to prevent the disease from developing into gynecological cancer: fractional pelvic external radiation therapy (EBRT) and vaginal brachytherapy (BRT) in session (Williamson et al., 2021). Thanks to adjuvant radiation therapy, it has been found to increase survival in gynecologic cancer cases. However, just like chemotherapy, radiation therapy can have adverse effects. For example, in one study, approximately 30% of women with endometrial cancer had acute diarrhea due to gastrointestinal toxicity after radiation therapy (Viswanathan et al., 2014). On the other hand, some clinical observations have observed permanent or transient redness, pain, and dry skin after radiation therapy. It can sometimes form in infected skin wounds after exposure to radiation (Baines et al., 2017). Long-term radiotherapy has detected cases of dermatitis and increased fibrosis (Bray et al., 2016).

New approaches have emerged in the treatment of the disease due to the serious side effects of traditional approaches to gynecological cancer treatment. Alternative therapies have been developed to reduce side effects, lower doses, and keep patients away from their daily routines. These include targeted therapy, hormone therapy, and immunotherapy (Ngu et al., 2022).

 In the treatment of gynecological cancers, hormones are used as a therapeutic auxiliary or post-surgical adjuvant effect: selective estrogen receptor modulators, aromatase inhibitors, gonadotropin-release hormone analogs (GnRHa), and progestogens (Kobori, 2022). For example, gonadotropins, follicle-stimulating hormone (FSH), luteinizing hormone (LH), progesterone, androgens, insulin-like growth factor-I (IGF-I), and estrogens play a role in the development of ovarian cancer. These hormones can be successfully used in the treatment of ovarian cancer (Wang et al., 2020a; Song et al., 2020a; Li et al., 2021; Kobori, 2022; Matsuoka et al., 2022). The human papillomavirus (HPV) is one of the most important causes of cervical cancer. It has been found to multiply viral DNA via progesterone, causing cervical cancer in the host (Oh et al., 2009; James et al., 2020). This information indicates that hormones play an important role in the treatment of gynecologic cancers. Another new approach for treatment is immunotherapy. The aim of immunotherapy is to destroy cancer cells by using a donor system (Pakish and Jazaeri, 2017). Immunity checkpoint inhibitors, T-cell transfer therapy, and stimulation of nonspecific immunity are used in the treatment of gynecological cancers. However, there is still no fully defined application for the treatment of gynecological cancers (Ngu et al., 2022). Currently, pembrolizumab, a control-point inhibitor (anti-PD-1 monoclonal antibiotic), is approved for treatment of some women with advanced endometrial cancer to prevent the progression or recurrence of the disease after chemotherapy (Pakish and Jazaeri, 2017; Jiang et al., 2023).

It is currently used in the new generation of targeted medicines for the treatment of gynecological cancers. Thanks to recently evolved and more knowledgeable cancer biology, valuable paths have been laid in the development of targeted treatments (Ngu et al., 2022). Angiogenesis and angiogenetic factors are required for the growth, nutrition, and invasion of cancer cells, for example (Yetkin-Arik et al., 2021). Bevacizumab, a recombinant monoclonal antibody targeting vascular endothelial growth factor (VEGF), which plays a role in angiogenesis, is used in the treatment of ovarian and cervical cancer (Yetkin-Arik et al., 2021). Nowadays, standard targeted gynecologic cancer therapy is a system that, independently of standard therapies and their cytotoxicity, inhibits DNA replication and mitosis, thereby interfering only with a single molecule to activate or deactivate certain signal pathways (Crusz and Miller, 2020). Targeted treatments are of two types. The first is small molecules that enter the cytoplasm and affect targets such as tyrosine kinases. These molecules specifically target the PI3K/AKT/mTOR pathways, which are involved in apoptosis and cell proliferation (Peng et al., 2022). For example, pazopanib is a nonspecific tyrosine kinase inhibitor and inhibits the platelet-derived growth factor receptor, primarily VEGF, thereby increasing survival in breast cancer (Pignata et al., 2015). On the other hand, molecules involved in DNA repair mechanisms and poly (ADP-ribose) polymerase (PARP) inhibitors are the most commonly used target molecules in the treatment of gynecologic cancers (Crusz and Miller, 2020). The second are monoclonal antibodies that bind to ligands or receptors on the cell surface and do not penetrate the cell. Monoclonal antibodies kill cancer cells through cytotoxicity. Currently, the most commonly used monoclonal antibodies include VEGFR antibody, bevacizumab, anti-programmed cell death-1 (PD-1) antibody, and pembrolizumab (Lee et al., 2023).

In the treatment of gynecological cancers, the disease is more complicated in other parameters than the difficult, restrictive, and side effects of therapies. The most critical phenomenon that complicates treatment is multidrug resistance (MDR) (Sabnis et al., 2021; Vemula et al., 2023). Chemoresistance reduces survival, especially in metastatic cancers (Tam et al., 2021). Numerous studies in the literature indicate that autophagy has a significant effect on chemoresistance development in cancer cells in two different ways. The first is primary/intrinsic resistance, and the other is secondary/acquired resistance (Amaral et al., 2019). Intrinsic drug resistance does not end completely, although there is a decrease in the size of the target lesion, often due to heterogeneous morphologies of cancer cells. This causes the cancer to become metastatic. Clinical trials to eliminate drug resistance have unfortunately failed (Amaral et al., 2019; Vemula et al., 2023). Apart from the heterogeneous cell structure, which is one of the biggest causes of the development of MDR or chemoresistance, unstable genes, polymorphisms, gene mutations, and modifications also cause drug resistance (Sabnis et al., 2021; Tam et al., 2021; Vemula et al., 2023). Developing resistance to treatment is actually a survival mechanism developed by cancer cells. Genetic and epigenetic predispositions, drug-metabolizing systems, metabolic enzymes, and drug efflux pump pathways of gynecological cancer patients are other parameters that are effective for cellular survival and the development of drug resistance (Amaral et al., 2019). Drug resistance can develop through ATP-binding cassette (ABC) and P-glycoprotein (P-GP)-mediated pathways when MDR is approached from a molecular point of view (Arain et al., 2021). In addition, decreased activation of pro-apoptotic protein members BAX and Fas and overexpression of anti-apoptotic proteins such as Bcl-XL, Bcl-2, and Mcl-1 trigger the mechanism of drug resistance in cancerous cells (Vemula et al., 2023). On the other hand, fibroblasts, endothelial cells, lymphoids, myeloids, and immune cells are cellular structures that play a role in the development of drug resistance. In addition, hypoxic conditions, pH of the tumor microenvironment, extracellular matrix, interleukin-6, B-cell-activating factor, VEGF, and PDGF-R affect the development of drug resistance (Tam et al., 2021). In addition, chemoresistant cells develop due to increased activation of MAPK/P13K/mTOR signaling pathways, which regulate cell survival and tumor growth from molecular pathways, translocation from the cytoplasm to the nucleus as a result of phosphorylation of nuclear factor kappa b (NFKB), and inhibition of the p53 tumor suppressor gene (Tam et al., 2021; Vemula et al., 2023).

## 3 Molecular mechanisms of coumarins in gynecological cancers

As expected, gynecologic cancers tend to appear by means of a coalescence of several factors and molecular-level metabolism rather than a single cellular process. The viability, proliferation, invasion, and metastasis of these cancerous cells are directly related to a network of complex signaling pathways and various molecular mechanisms. The results of a large body of experimental studies indicate the considerable potential of natural compounds in lessening the development of gynecologic cancers. Such compounds not only act directly on the cellular mechanisms of cancer cells and impede their progress but also render these cells more vulnerable to standard chemotherapy, and overcome the chemoresistance phenomenon (Farrand et al., 2014; Wang et al., 2020b).

**FIGURE 6:**
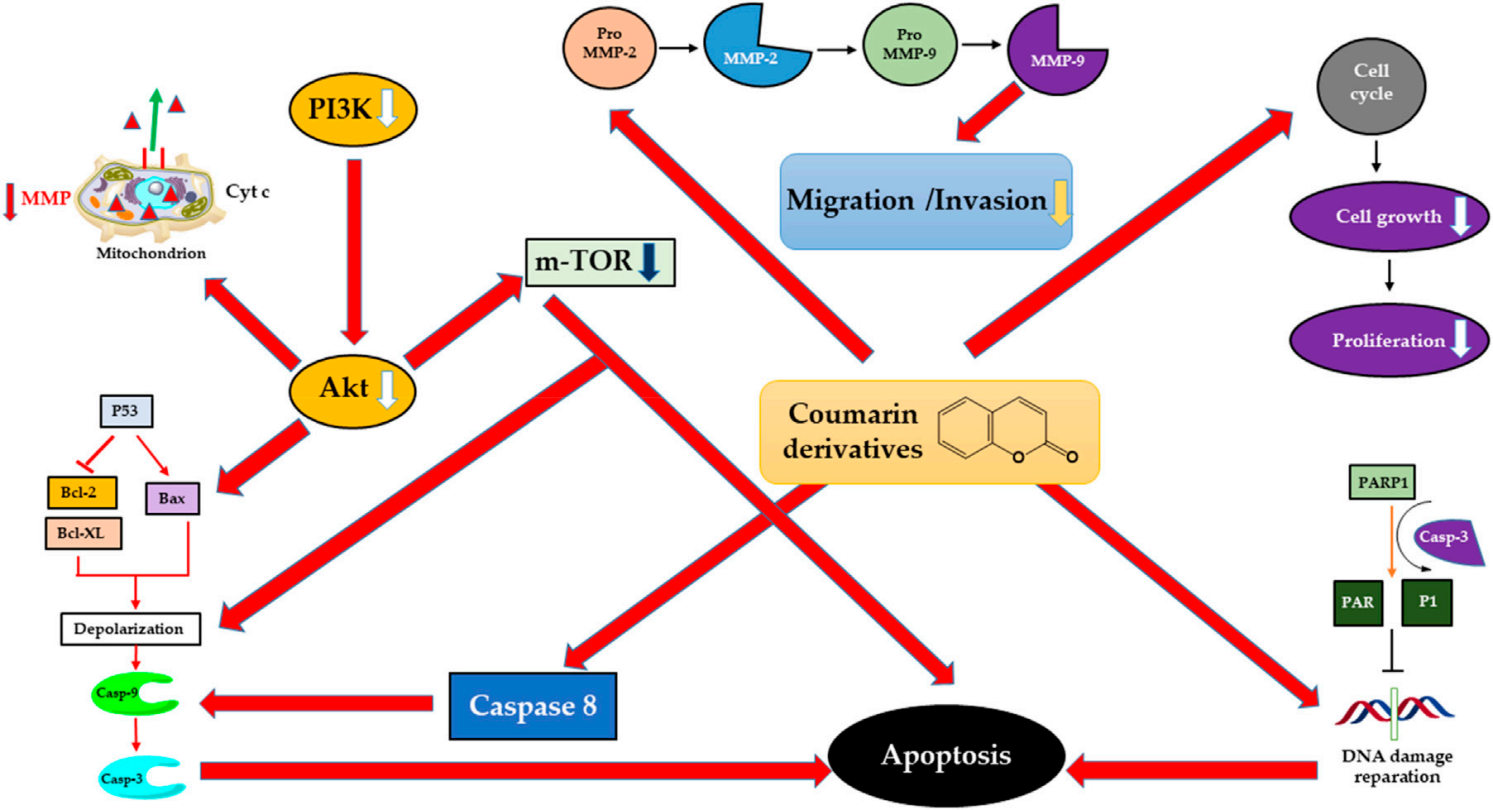
Mechanisms of action of natural coumarin-derived compounds on gynecological cancer cells.

Data from a diverse range of studies state the noteworthy potential of coumarins in the effort against proliferation, invasion, and metastasis of ovarian, cervical, or endometrial cancers (De La Cruz-Concepción et al., 2021). Coumarins have a significant impact on the cell viability or proliferation of gynecologic tumor cells by halting the cell cycle progression via the upregulation of negative regulators or by inducing cell death through enhancing the expression of proapoptotic proteins and downregulating the expression of proteins responsible for antiapoptosis and angiogenesis. Alternatively, coumarins have been reported to impede the migration and invasion of gynecologic tumor cells, primarily via the suppression of matrix metalloproteinase -2 and -9 expression and activity. In addition, various signaling pathways such as PI3K/AKT have been demonstrated to play a pivotal role in the antitumor effect of coumarins. Additionally, they are efficient in overcoming the chemoresistance barrier of some widely used chemotherapeutics (Wu et al., 2020; De La Cruz-Concepción et al., 2021). In summary, coumarins have the potential to act on cell proliferation, angiogenesis, and apoptosis of gynecologic cancer cells (Figure 6). A list of mechanisms through which coumarins conduct their anticancer potential on such cancers is given below (for an overview, see Table 3).

**TABLE 3:** Overview of anticancer mechanisms of natural coumarins in gynecological cancers.

Compound	Cell line	Mechanism	References
(+)-4′-decanoyl-cis-khellactone (+)-3′-decanoyl-cis-khellactone	HeLa SiHa C33A	Induce apoptosis Induce cell cycle arrest in the S/G2 phase	Jung et al. (2012)
4-methylumbelliferone	ES2 OV90	Decreases AKT phosphorylation Induces cell cycle arrest in the G2/M phase	An et al. (2020)
7-hydroxycoumarin (umbelliferone)	SKVCR	Induces cell cycle arrest in the G2/M phase	Wang and Wang (2021)
Angelicin	HeLa SiHa	Induces cell cycle arrest in the G1/G0 phase	Wang et al. (2019b)
Angelol-A	HeLa SiHa	Supresses the cell migration and invasion Downregulates the expression of MMP-2 Downregulates the ERK pathway	Ying et al. (2022)
Auraptene	A2780 HeLa	Inhibits the activities of MMP-2 and -9	Jamialahmadi et al. (2018)
Bergenin	HeLa	Upregulates Bax expression Downregulates Bcl-2 expression Induces cell cycle arrest in the G0/G1 phase Decreases STAT3 protein phosphorylation	Shi et al. (2019)
Daphnetin	A2780	Decreases p-Akt, and p-mTOR expression levels Increases p-AMPK expression	Fan et al. (2021)
Dicoumarol	SKOV-3	Decreases PDK1 kinase activity Weakens mitochondrial membrane potential Induces apoptosis	Zhang et al. (2017)
	A2780	Increases cleaved caspase and its substrate-cleaved PARP Induces apoptosis Decreases p-PDHA1 level	
Esculetin	HO-8910	Induces the upregulation of the Bax/Bcl-2 ratio Induces the cleaved caspase-3 and −9 expression	Chang et al. (2018)
	HEC-1B Ishikawa	Induces G2/M arrest and apoptosis Increases the cleaved caspase-3 and PARP expression Downregulates the expression of Bcl-XL and XIAP	Jiang et al. (2021)
	SKOV-3	Upregulates Bax, cytochrome C, and cleaved caspase-3 and -9 Increases the expression of Bcl-2 Downregulates the expressions of MMP-2 and -9 Induces cell cycle arrest in the G0/G1 phase Reduces the expressions of cyclin D1 and cyclin E, together with CDK4 and CDK2	Yin et al. (2023)
Fraxetin	End1/E6E7 VK2/E6E7	Decreases the phosphorylation of p38, JNK, and ERK1/2	Ham et al. (2024)
Osthole	JEC KLE Ishikawa	Increases the expressions of Bax, cleaved caspases-3 and -9, and PARP	Liang et al. (2021)
	A2780 OV2008	Inhibits the activities of MMP-2 and -9 Decreases the expressions of MMP-2 and -9 Inhibits the protein expressions of cyclin B1 and CDK2 Induces the cell cycle arrest in the G2/M phase	Jiang et al. (2016)
	ES2 OV90	Suppresses the PI3K signaling pathway Decreases the levels of p-ERK1/2, p-JNK, and p-p38 Inactivates cyclin D1 Induces G1 accumulation	Bae et al. (2021)
	SiHa CaSki	Inactivates inactivation of the PI3K/AKT pathway	Su et al. (2019)
Praeruptorin A	HeLa	Decreases MMP-2 expression Increases TIMP-2 expression Suppresses ERK1/2 phosphorylation	Wu et al. (2018)
Praeruptorin B	HeLa	Inhibits the expression and transcriptional activity of MMP-2 and -9 Suppresses the phosphorylation of the PI3K/AKT pathway	Hung et al. (2019)
Pulchrin A	CAOV-3	Induces apoptosis Activates procaspases 3 and 9 and cleaved caspases 3 and 9 Downregulates Bcl-2 expression Upregulates Bax expression Induces cell cycle arrest in the G0/G1 phase	Nordin et al. (2016)
Scopoletin	HeLa	Increases Bax expression Downregulates Bcl-2 expression Induces the cleavage of caspase-3, -8, and -9 Inhibits cell proliferation by suppressing phosphorylation Inhibits the PI3K/AKT signaling pathway Generates the arrest of the cell cycle in the G2/M phase	Tian et al. (2019)
Sinkiangenol E	HeLa	Increases p-ERK/ERK, p-JNK/JNK, and p-p38/p38 Induces cell cycle arrest in the G0/G1 phase	Wang et al. (2020)
Visnagin	HeLa	Decreases the gene expressions of PI3K, AKT, and mTOR proteins	Qi et al. (2022)

### 3.1 The effect on apoptosis-related proteins of coumarins

Apoptosis is an intricate cascade of programmed cell death, involving a complex network of enzymes and proteins. The caspase enzyme family is well-known to play a pivotal role in the process. Among them, caspases -2, -8, -9, and -10 take part in the initiation of apoptosis, while caspases -3, -6, and -7 are responsible for the execution step by hydrolyzing a wide array of structural and functional proteins, including PARP (poly-ADP-ribose polymerase). Within this group, the Bcl-2 (B-cell lymphoma-2) family occupies a significant place in apoptosis, containing proteins responsible for both pro- and anti-apoptosis. Proapoptotic members include Bax, Bad, Bak, Bim, Bid, and PUMA (p-53 upregulated modulator of apoptosis). They take part in the mitochondrial outer membrane permeabilization (MOMP), a key step in the initiation of apoptosis. On the other hand, Bcl-2 is a potent inhibitor of apoptosis and suppresses the liberation of cytochrome c, a proapoptotic factor (Assaf et al., 2022; Shi et al., 2024).

Scopoletin is a major coumarin found in various plants but mainly extracted from *Scopolia* spp. In HeLa cervical cancer lines, scopoletin treatment was found to considerably upregulate Bax expression, while it downregulated the expression of Bcl-2. In addition, the cleavage of caspases-3, -8, and -9, together with PARP was also observed to escalate in HeLa cells, following scopoletin treatment (Tian et al., 2019).

Esculetin, 6,7-dihydroxycoumarin, another important coumarin found in many plant sources, induces a dose-dependent increase in the expression level of cleaved caspase-3 and PARP in both HEC-1B and Ishikawa human endometrial cancer lines. The same study suggested that esculetin downregulated the expression of the apoptotic proteins Bcl-XL and XIAP in these cell lines. The same downregulation was also observed *in vivo* (Jiang et al., 2021). In another study on human ovarian carcinoma cell lines (HO-8910), esculetin was observed to dose- and time-dependently increased the Bax/Bcl-2 ratio. It also prompted an increase in the expression of cleaved caspase-3 and -9 (Chang et al., 2018). A similar study on a different human ovarian cancer line (SKOV-3) revealed that esculetin treatment dose-dependently upregulated the apoptosis-linked proteins Bax, cytochrome C, and cleaved caspases-3 and -9. In addition, esculetin induced an elevation in the expression of Bcl-2, leading to an increase in the Bax/Bcl-2 ratio. In this manner, the increase in Bax expression and reduction in Bcl-2 expression promotes mitochondrial depolarization, an indication of proapoptotic characteristic in ovarian cancer cells (Yin et al., 2023).

Osthole, a prenylated coumarin derivative obtained from the fruits of *Cnidium monnieri* (L.) Cusson was tested on three different endometrial cancer cell lines (JEC, KLE, and Ishikawa). The molecule was shown to increase the expression of Bax, cleaved caspases −3 and −9, and PARP. Furthermore, the study revealed that the activity of caspase-9 was significantly elevated during osthole treatment (Liang et al., 2021).

Jakubowicz-Gil et al. investigated the mechanism of cell death caused by imperatorin and cisplatin in the HeLa cell line. Imperatorin has been determined to be a potent autophagy inducer, whereas cisplatin mainly induces apoptosis and necrosis. When imperatorin and cisplatin were coadministered to HeLa cells, autophagy was observed and associated with the presence of the cleaved form of microtubule-associated protein 1 light chain LC3–LC3II. The expression of the heat shock proteins Hsp27 and Hsp72 was also inhibited after the co-treatment of drugs (Jakubowicz-Gil et al., 2012). In a different study, it was reported that 50 µM quercetin and imperatorin produced the highest proportion of apoptotic cells when applied for 48 h. When given concurrently, apoptosis was elicited more potently than with a single medication. These findings were validated by molecular experiments, which also showed increased caspase activity and decreased expressions of Hsp27 and Hsp72. There were no discernible alterations in the expression of beclin-1, and autophagy was not observed (Bądziul et al., 2014).

In another research, the C-4-II cell line (HPV-18 positive cervical cancer cell) was used to study the functionalization of antisense oligonucleotides using the photocross-linking reagent 4,5′,8-trimethoxypsoralen. The IC50 value was 16 nM. The mechanism has been determined to be p53-induced apoptosis (Yamayoshi et al., 2003).

Dicoumarol has been demonstrated to strongly decrease PDK1 kinase activity, change glucose metabolism from aerobic glycolysis to oxidative phosphorylation, increase ROS levels, weaken mitochondrial membrane potential, trigger apoptosis, and decrease cell number in SKOV-3 cells. In A2780 ovarian cancer cells, it was also found that cleaved caspase and its substrate-cleaved PARP increased, p-PDHA1 level decreased, and apoptosis was promoted (Zhang et al., 2017).

Two natural khellactone compounds, (+)-4′-decanoyl-cis-khellactone and (+)-3′-decanoyl-cis-khellactone, were isolated from the rhizome of *Angelica purpuraefolia* Chung. These pyranocoumarins were examined for their effects on the HeLa, SiHa, and C33A cervical cancer cell lines. The findings demonstrated that at concentrations of less than 10 μg/mL, (+)-4′-decanoyl-cis-khellactone and (+)-3′-decanoyl-cis-khellactone inhibited the development and proliferation of cancer cells, and at concentrations greater than 50 μg/mL, they triggered apoptosis. It has been determined that these compounds stop the cell cycle progression of cervical cells in the S/G2 phase and cause caspase-dependent apoptosis (Jung et al., 2012). Interestingly, an earlier study found that these khellactones showed no substantial cytotoxicity (IC50 > 100 μM) in the SKOV-3 cancer cell line (Chung et al., 2010).

Nordin et al. isolated a new coumarin derivative from the natural product *Enicosanthellum pulchrum* (King) Heusden, which was deduced as pulchrin A. Pulchrin A was evaluated for its ability to induce apoptosis in CAOV-3 and SKOV-3 cells. The results showed that pulchrin A exhibited stronger cytotoxic effects on CAOV-3 cells at a concentration as low as 22 μM after 24 h of exposure and was more effective than the control drug cisplatin. Apoptosis was initiated via the intrinsic pathway, which involved the activation of procaspases 3 and 9, along with cleaved caspases 3 and 9, and finished at the executioner pathway, where DNA laddering occurred. Additionally, the Bcl-2 protein was determined to be downregulated, the Bax protein was upregulated, and CAOV-3 was disrupted in the G0/G1 phase of the cell cycle (Nordin et al., 2016).

### 3.2 Effect on the inhibition of extracellular matrix (ECM) degradation of coumarins

Any modifications in the integrity of the extracellular matrix (ECM) might be a possible reason for tumor development, and eventually metastasis. Matrix metalloproteinase (MMP) enzymes constitute a large family of zinc-dependent endopeptidases and instigate ECM degradation, which will bring about angiogenesis, proliferation, and invasion of cancer cells. Among the members of the MMP family, MMP-2 and MMP-9 are known to play a direct role in tumor metastasis. The expression of proteins and mRNA of MMP-2 is significantly elevated in human cervical cancer, while the release of MMP-9 induces angiogenesis and metastasis of these tumor cells (Kato et al., 2002; Tanaka et al., 2019). Thus, suppression of these MMPs and ECM degradation is thought to be an effective way of dealing with gynecologic cancer progression.

Yin et al. studied the inhibitory effect of esculetin on MMP-2 and MMP-9 enzymes in SKOV-3 human ovarian cancer cells by Western blotting. Their results indicated that esculetin treatment caused significant downregulation in the expressions of MMP-2 and MMP-9 in those cells (Yin et al., 2023). In a similar study, auraptene, a natural coumarin mainly obtained from *Citrus spp*., was shown by gel zymography to dose-dependently inhibit the activities of both MMP-2 and MMP-9 in both human ovarian cancer (A2780) and cervical cancer (HeLa) cell lines. It was demonstrated that after 24 h of exposure, it significantly reduced the viability of HeLa cells with an IC50 of 47.93 μM. Auraptene, at concentrations ranging from 50–100 μM for 6–24 h, was found to reduce migration and invasion capacity (Jamialahmadi et al., 2018).

Another natural coumarin, angelol-A, which is isolated from the roots of *Angelica pubescens* Maxim, was reported to have a significant suppressing effect on invasion and angiogenesis in different human cervical cancer cell lines (SiHa and HeLa). MMP-2 expression in those cell lines was found to be downregulated by angelol-A in a concentration-dependent manner. Conversely, the molecule did not promote the same effect on MMP-9 (Ying et al., 2022). Jiang et al. investigated the same effect of osthole in ovarian cancer lines (A2780 and OV2008). Their results demonstrated that osthole concentration-dependently blocked the activities of MMP-2 and MMP-9. At the same time, dramatic decreases were reported in the expression of these enzymes, following the treatment (Jiang et al., 2016).

Similar findings were also reported for a different subclass of coumarins. Praeruptorin A, a pyranocoumarin, was isolated from the roots *Peucedanum praeptorum* Dunn. The molecule was shown in HeLa cells to significantly diminish MMP-2 expression. In addition, the expression of tissue inhibitor metalloproteinase-2 (TIMP-2) was upregulated. These results manifest the inhibitory effect of praeruptorin A on ECM degradation (Wu et al., 2018). Likewise, another pyranocoumarin from the same plant, praeruptorin B was reported to inhibit the expression and transcriptional activity of MMP-2 and MMP-9 in HeLa cells (Hung et al., 2019).

### 3.3 Effect on the PI3K/AKT mTOR signaling pathway of coumarins

PI3K/AKT/mTOR is a signaling pathway of key importance, participating in cell proliferation, cell differentiation, angiogenesis, apoptosis, and metastasis. The pathway also takes a considerable part in the maintenance of the cell cycle. PI3K (phosphatidylinositol kinase) is responsible for the activation of a group of protein kinase enzymes, including AKT (protein kinase B), which is a major target site for PI3K (Assaf et al., 2022). AKT is known to maintain protein expression associated with MMPs, and it also has the ability to activate cyclin-dependent kinase (CDk)-4 and -2, leading to a complete cell cycle. AKT also takes part in apoptosis by inactivating Bax and particular members of the caspase family and in blocking the release of cytochrome C and other mitochondrial apoptosis-inducers, resulting in an antiapoptotic nature. mTOR is also a protein kinase responsible for protein translation and further leading to protein synthesis. It is also known to be an AKT-regulated downstream target (Yu and Cui, 2016; Wee and Wang, 2017; Assaf et al., 2022). Furthermore, previous studies indicated that activation of the PI3K/AKT/mTOR signaling pathway is directly linked to the progression of gynecologic cancers (Bai et al., 2015). In this context, the inhibition of the PI3K/AKT/mTOR signaling pathway remains to be an important target in the treatment of those cancers.

Fan et al. used IOSE8C, A2780, SKOV-3, and OVCAR-8 cells to investigate the anticancer effect of daphnetin in ovarian cancer. The outcomes showed that, particularly in A2780, daphnetin administration dramatically reduced the number of colonies generated in a dose-dependent manner. In A2780 cells treated with this natural coumarin, p-Akt and p-mTOR expression levels were decreased, but p-AMPK expression levels were increased. The findings suggest that in ovarian cancer cells, aberrations in the AMPK/Akt/mTOR pathway are linked to daphnetin-induced autophagy (Fan et al., 2021).

Bae et al. studied the inhibitory effect of osthole on two different lines of ovarian cancer cells (ES2 and OV90) and reported that the molecule significantly suppressed tumor progression in those cell lines. The further mechanistic evaluation revealed the significance caused by osthole on the PI3K signaling pathway (Bae et al., 2021). The effect of the same molecule on human cervical cancer was studied by a different research group on both SiHa and CaSki cells. Their findings suggested that osthole caused the inactivation of the PI3K/AKT pathway in these cells. Interestingly, they showed that osthole induced a similar effect on cell lines that possess resistance against cisplatin, providing a potential reversal of chemoresistance in cervical cancer treatment (Su et al., 2019).

Praeruptorin B suppresses the phosphorylation of the PI3K/AKT signaling pathway, generating a considerable reduction in TPA (12-O-tetradecanoylphorbol-13 acetate)-induced cell invasion in different human cervical cancer lines (SiHa and HeLa) at various concentrations (Hung et al., 2019). As expected, these results suggest that praeruptorin B reduced the metastasis and invasion potential in those cell lines. Similarly, the anticancer potential of another natural coumarin, scopoletin, was investigated on HeLa cells. It was found that scopoletin treatment at increasing concentrations blocked cell proliferation by suppressing phosphorylation and inhibiting the PI3K/AKT signaling pathway (Tian et al., 2019).

4-methylumbelliferone, a coumarin derivative isolated from various plant sources and previously reported to possess potential anticancer effects, was tested on epithelial ovarian cancer cell lines (ES2 and OV90). The phosphorylation levels of proteins in the PI3K/AKT pathway were measured by Western blotting analysis in these cell lines. The findings indicated a significant reduction in AKT phosphorylation, subsequent to 4-methylumbelliferone treatment. Furthermore, the combinatory treatment of 4-methylumbelliferone and known PI3K inhibitors generated a synergistic effect and brought about a more dramatic decrease in phosphorylation levels, compared to the 4-methylumbelliferone treatment alone (An et al., 2020).

### 3.4 Effect on the MAPK signaling pathway of coumarins

The mitogen-activated kinase (MAPK) signaling transduction plays a direct role in cancer physiology. Any imbalance or over-activation of this pathway might affect cell proliferation, differentiation, or invasion. MAPK enzymes include JNK (c-Jun N-terminal kinase), p38, and ERK (extracellular signaling-regulated kinase). Activation of this pathway leads to cell proliferation, apoptosis, and invasion of tumor cells, along with resistance to some chemotherapeutics in use. A broad array of cancers is evoked via the MAPK pathway, constituting a potential target in cancer therapy (Akhtar et al., 2020; Shi et al., 2024).

Wang et al. studied the role of sinkiangenol E, a sesquiterpene coumarin from the resin of *Ferula siknkiangensis* K.M.Shen, in the MAPK pathway in HeLa cells. Their results indicated that the treatment significantly increased the ratios of phosphorylated levels of MAPK proteins over their total protein expressions. p-ERK/ERK, p-JNK/JNK, and p-p38/p38 were reported to significantly elevate in these cells (Wang et al., 2020).

Angelol-A, a coumarin isolated from the roots of *A. pubescens* Maxim, was tested on human cervical cancer lines (HeLa and SiHa). The treatment prompted the downregulation in the ERK pathway. Further investigation revealed that the cotreatment of angelol-A with a known ERK1/2 inhibitor generates a synergistic inhibitory activity. Overall results indicated that angelol-A treatment suppresses metastasis and invasion of cervical cancer cells through the ERK pathway (Ying et al., 2022).

Ham et al. investigated the effect of fraxetin, a methoxy-substituted coumarin derivative isolated from *Fraxinus rhynchophylla* Hance, different endometriotic epithelial cell lines (End1/E6E7, and VK2/E6E7). The results displayed that the treatment induced a gradual reduction in p38 phosphorylation, while the phosphorylation of both JNK and ERK1/2 was also gradually declined. Notably, despite the downregulation in p-ERK1/2 expression by the combined treatment of fraxetin with a known inhibitor, no synergistic interaction was observed (Ham et al., 2024).

In human ovarian cancer cell lines (ES2 and OV90), osthole treatment prompted a significant, dose-dependent decline in the levels of phosphorylated MAPK signaling pathway proteins, including p-ERK1/2, p-JNK, and p-p38 (Bae et al., 2021). Praeruptorin A was found to significantly suppress ERK1/2 phosphorylation at different concentrations in HeLa cells, revealing a direct participation in the blocking of cell migration and metastasis. However, the treatment did not induce any change in the phosphorylation of JNK or p38 (Wu et al., 2018).

Visnagin, which has a furanocoumarin structure, caused apoptosis in the HeLa cell line at 15 µM and 25 μM, and at the same doses, it decreased the gene expressions of PI3K, AKT, and mTOR proteins, as well as the extracellular cell proliferation signaling pathway MAPK pathway, ERK1/2, p38, and JNK1/2 proteins (Qi et al., 2022).

### 3.5 Effect of coumarins on cell cycle

To ensure that the hereditary data are replicated and distributed throughout the cell faultlessly, the cell cycle is monitored by an intricate complex of different mechanisms. Cell division and the duplication of cell components consist of four subsequent phases: growth phase 1 (G1), synthesis phase (S), growth phase 2 (G2), and mitosis (M). The regulation of this process is maintained by cyclins and cyclin-dependent kinases (CDKs), also known as regulatory proteins. In relation to this, any malfunction in the cell cycle might lead to tumor growth. Such cancerous cells might no longer have control over the inhibitory mechanisms (Bailon-Moscoso et al., 2017), resulting in the upregulation of the mentioned control systems and eventually dysregulated cell division. Cell cycle arrest is considered to be a key concept in cancer treatment; therefore, the search for cell cycle inhibitors keeps on continually. Mitotic spindles, microtubules, and proteins linked to DNA replication constitute possible targets for such inhibitors Microtubules are major constituents of mitotic spindles, which are directly responsible for the mitotic activity. Molecules with the ability to arrest the cell cycle prompt the upregulation of microtubules or suppress their aggregation (Akhtar et al., 2020; Wu et al., 2020).

Wang et al. investigated the mechanisms behind 7-hydroxycoumarin’s action on ovarian cancer cell lines that are resistant to cisplatin (SKVCR cell line). A caspase-linked apoptotic pathway caused the induction of cell death, which resulted in a decrease in cell proliferation. The administration of 7-hydroxycoumarin furthermore resulted in the G2/M stage cancer cell cycle arrest by downregulation of regulatory protein expressions that facilitate mitotic entrance (Wang and Wang, 2021).

Yin et al. performed an extensive investigation into the anticancer potential of esculetin in SKOV-3 human ovarian cancer cells. Their results indicated that the molecule induced cell cycle arrest in the G0/G1 phase. It was also observed to significantly reduce the expressions of cyclin D1 and cyclin E, together with CDK4 and CDK2 in the ovarian cancer cells (anti-ovarian) (Yin et al., 2023). Sinkiangenol E, a newly described sesquiterpene coumarin isolated from the resin of *Ferula sinkiangensis*, was tested in HeLa cells. The results showed that treatment with this molecule dose-dependently arrested the cervical cancer cell cycle in the G0/G1 phase (Wang et al., 2020). Analogously, 4-methylumbelliferone diminished the cell proliferation of epithelial ovarian carcinoma cell lines (ES2 and OV90) through cell cycle arrest. The molecule was observed to arrest the cell cycle in the G2/M phase (An et al., 2020). In the same two cell lines, the effect of osthole treatment as a potential anticancer mechanism was investigated. At increasing doses, the treatment was reported to downregulate the proliferation of these cell lines. It was also observed that osthole inactivated cyclin D1 through the suppression of its phosphorylation. At lower doses, osthole prompted G1 accumulation, while at higher doses, it induced G2 accumulation in both OV90 and ES2 cancer cell lines. Interestingly, further investigation showed that the treatment did not affect normal cells, but targeted cancer cells (Bae et al., 2021). Another study regarding osthole and its anticancer potential on different ovarian cancer cell lines (A2780 and OV2008) was carried out by Jiang et al. They reported that osthole treatment on these cells caused the inhibition of protein expressions of cyclin B1 and CDK2. This resulted in the induction of cell cycle arrest in the G2/M phase (Jiang et al., 2016). The effect of scopoletin was also tested in HeLa cells, and it was found that the molecule generated the cell cycle arrest in the G2/M phase. The percentage of HeLa cells in the G2 phase was significantly elevated (Tian et al., 2019). In a similar study, angelicin was evaluated for its antitumor effects in cervical cancer cell lines. Angelicin reduced HeLa and SiHa cell growth at IC30 (27.8 µM and 36.6 µM, respectively) by interrupting the cell cycle at the G1/G0 phase, as well as other malignant characteristics such as colony formation, tumor formation, migration, and invasion (Wang et al., 2019b).

Bergenin is an isocoumarin that can be isolated from *Bergenia* species. Its effect on the HeLa cervical cancer cell line has been investigated by Shi et al.; this isocoumarin caused apoptosis, upregulated the expression of Bax, and downregulated the expression of Bcl-2. Bergenin additionally resulted in cell cycle arrest at the G0/G1 phase and reduced STAT3 protein phosphorylation (Shi et al., 2019).

## 4 Coumarin-based hybrids as potential anticancer agents for gynecologic cancers

Multi-target strategies occupy a pivotal position in the treatment of cancer, given that single-target therapies frequently lose efficacy over time. Studies such as the coumarin-based hybrid molecules developed by Singh et al. (2019) have the potential to ensure that treatment is effective across a broad spectrum by affecting multiple biological pathways. By interfering with the various growth and survival pathways of cancer cells, these molecules seek to circumvent the resistance mechanisms of cells (Singh et al., 2019). Coumarin-based hybrid molecules have been developed to address this issue. Singh et al. (2019) highlight the limitations of single-target therapies in cancer treatment due to the multifactorial nature of the disease, suggesting that multi-target therapies may be more effective. This concept is consistent with the findings of the study by Cao et al. (2016), where a novel coumarin derivative was synthesized and tested for its antiproliferative properties against a wide range of tumor cells. Cao et al. have synthesized a series of new coumarins and tested them for their antiproliferative properties against a wide range of tumor cells. Among them, 5-chloro-n-(2-methoxy-5-(methyl (2-oxo-2Hchromen-4-yl) amino) pentanamide exhibited strong antitumor proliferation ability with an IC50 value ranging from 3.5 to 31.9 nM. The compound induced the cell cycle arrest of human ovarian cancer cells (A2780s and A2780/T) in the G2/M phase and promoted the apoptosis of the cells. At a concentration of 30 nM, the compound induced significant arrest of A2780 S and A2780 T cells in G2/M at 37.07% and 29.94%, respectively, in G2/M. At a higher concentration of 300 nM, the percentage of cells in G2/M increased to 90.23% for A2780/T and 76.9% for A2780s. The IC50 value of the compound for A2780S cells is 33.7 ± 12.4 nM. This concentration inhibits cell proliferation by 50% in A2780S cells. In addition, it showed significant antiproliferative activity against tumor cells overexpressing P-gp or class III β-tubulin as well as multidrug-resistant cells. The compound, a synthetic 4-substituted coumarin derivative, exhibits potent anticancer activity through inhibition of tubulin polymerization. It disrupts microtubule dynamics by interacting with the colchicine binding site on tubulin. Cao et al. (2016) demonstrated that synthetic 4-substituted coumarin derivative exhibited potent anticancer activity, inhibiting tubulin polymerization and disrupting microtubule dynamics through interaction with the colchicine binding site on tubulin. This mechanism resulted in cell cycle arrest at the G2/M phase and induced apoptosis in cancer cells. The compound demonstrated significant antiproliferative effects against a range of cancer cell lines, including those resistant to multiple drugs, indicating its potential as a promising anticancer agent (Cao et al., 2016). Furthermore, Cao et al. (2016) demonstrated the compound’s efficacy *in vivo* in inhibiting tumor growth without significant adverse effects, suggesting its potential for clinical use in cancer treatment. The findings of this study support the concept of developing multi-target therapies, such as coumarin-based hybrid molecules, to effectively target the complex mechanisms involved in cancer progression and overcome issues such as drug resistance.

Govindaiah et al synthesized and evaluated the anticancer activity of new 4,7-dihydroxycoumarin-based acryloylcyanohydrazone derivatives (8a-m) against HeLa cancer cell lines. The results showed that, with IC50 values ranging from 3.42 to 31.28 µM, the compounds exhibited good-to-excellent cytotoxicity. The effectiveness of the compounds varied depending on modifications to the phenyl ring. The addition of a cyano group to the hydrazone moiety was found to enhance the activity of compounds with a hydrazide–hydrazone skeleton. Among the tested compounds, 8h exhibited the highest activity. The impact of compound 8h on cell cycle progression and its ability to inhibit tubulin polymerization were examined. Experimental results indicate that compound 8h arrests the cell cycle at the G2/M phase and inhibits tubulin polymerization with an IC50 value of 6.19 µM. Molecular binding simulations confirm the experimental data and demonstrate that the compound forms strong hydrogen bonds with specific amino acids (ASN-101, TYR-224, ASN-228, and LYS-254) in the tubulin protein (Govindaiah et al., 2019).

In conclusion, the study by Cao et al. (2016) exemplifies the development of a coumarin derivative with potent anticancer properties that target multiple pathways involved in cancer cell proliferation and survival. This aligns with the concept of multi-target therapies advocated by Singh et al. (2019) to address the challenges posed by the multifactorial nature of cancer. Both Singh et al. (2019) and Cao et al. (2016) emphasize the importance of multi-target therapies in cancer treatment due to the complex and multifactorial nature of the disease. Singh et al. (2019) highlight the limitations of single-target therapies and suggest that targeting multiple receptors or signaling pathways simultaneously may be more effective. In contrast, Cao et al. (2016) demonstrate the efficacy of a novel coumarin derivative in inhibiting tumor cell proliferation by targeting tubulin polymerization and disrupting microtubule dynamics, ultimately leading to cell cycle arrest at the G2/M phase and apoptosis. Furthermore, Govindaiah et al. (2019) contribute to this discussion by synthesizing new coumarin derivatives and evaluating their anticancer activity against HeLa cancer cell lines. They identified compound 8h as the most potent, demonstrating significant cytotoxicity and the ability to induce cell cycle arrest at the G2/M phase by inhibiting tubulin polymerization. Overall, these studies collectively support the concept of developing multi-target therapies using coumarin-based hybrid molecules to effectively combat the multifaceted mechanisms involved in cancer progression. This offers promising avenues for future anticancer drug development.

Cancer is characterized by evading apoptosis. This is why targeting apoptosis in certain cancer cells is a widely used approach to developing appropriate chemotherapeutics. Singh et al. synthesized a series of coumarin-based hybrid molecules, including coumarin and nifurtimox, and evaluated them for their anticancer activity. Although less potent, nifurtimox showed cytotoxicity against ovarian cancer cell lines. RKS262, a coumarin derivative containing the 1-aminotetrahydrothiazine ring of nifurtimox, was the most potent of the nifurtimox analogs tested. Singh et al. conducted a study to investigate the antitumor potential of RKS262 in OVCAR-3 cells, which carry different mutations found in a variety of ovarian cancers. The study measured the antiproliferative effects of RKS262 and analyzed the expression profile of the pro-apoptotic and pro-survival Bcl-2 family proteins in ovarian cancer cells. The data showed that RKS262 treatment resulted in apoptosis in OVCAR-3 cells, directly correlating with the oncogene RAS. Importantly, the activity of RKS262 has been evaluated in an objective and unbiased manner. In the case of ovarian cancer, RKS262 showed strong cytotoxicity (Singh et al., 2011). MAP kinases have been implicated in studies investigating the mechanisms of anticancer treatment in ovarian cancer cells (Mansouri et al., 2003; Lange et al., 2010). However, activation of the key pro-apoptotic MAPKs P38 and SAP/JNK was not significantly associated with RKS262-induced cytotoxicity (Singh et al., 2011).

**FIGURE 7:**
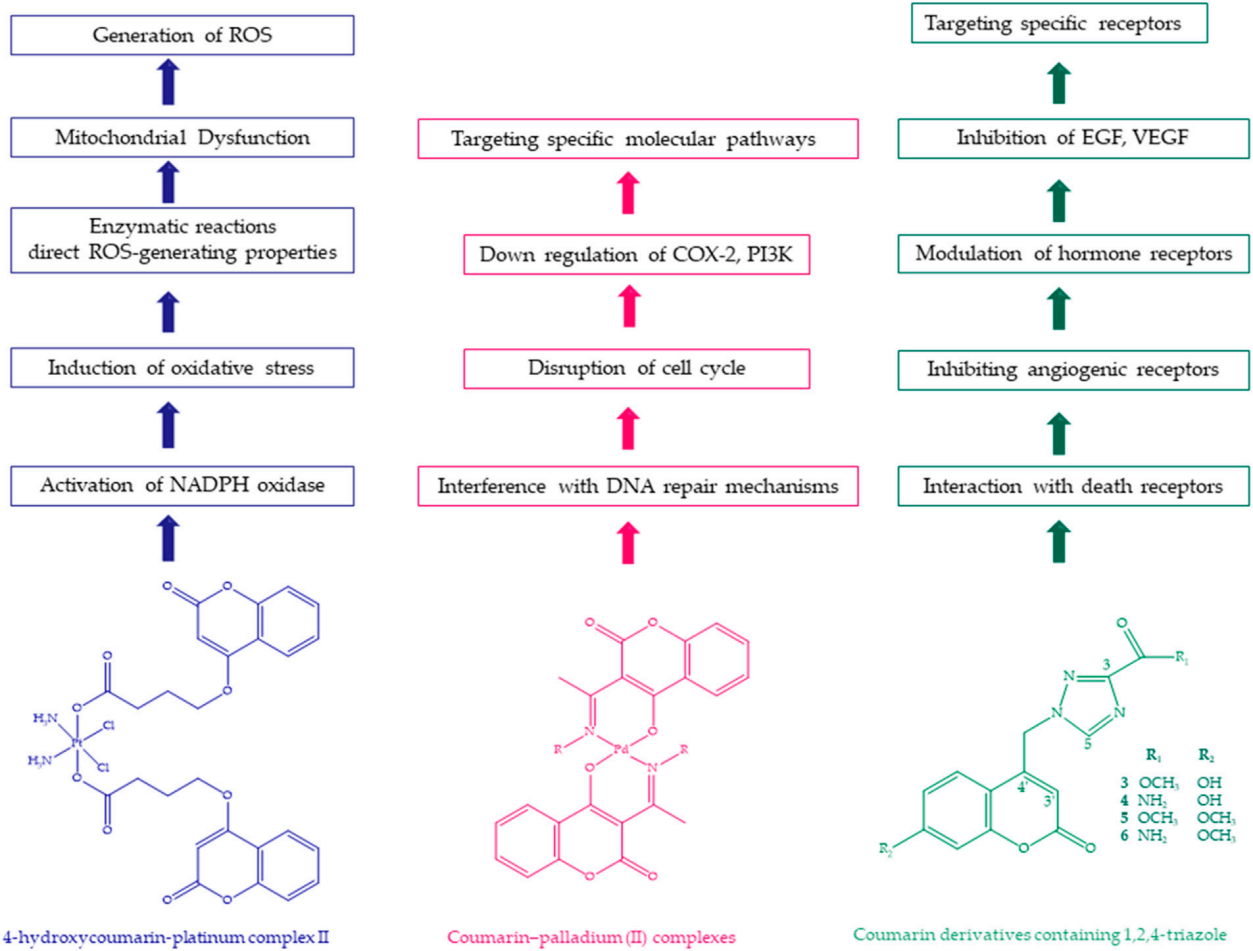
Structure and mechanisms of 4-hyydroxycoumarin–platinum complex II, coumarin–palladium (II) complex, and coumarin derivatives containing 1,2,4-triazole.

Avdović et al. synthesized novel coumarin–palladium (II) complexes (C1 and C2) and evaluated their effects on human cancer cells. Two new palladium (II) complexes containing bidentate coumarin ligands were synthesized and evaluated for their cytotoxicity. Compound C1 exhibited the most potent cytotoxic activity on all cell lines, except HeLa, in in vitro cytotoxicity tests on five different cancer cell lines. Compounds C1 and C2 were found to be pro-apoptotic in HeLa cancer cells. The experimental results showed an excellent correlation with RTK (receptor tyrosine kinase) inhibition. Furthermore, when compared to commercially available therapeutic standards, these compounds showed equivalent or superior inhibitory activity. The molecular binding studies showed that these complexes had a cytotoxic effect on human cancer cell lines and a pro-apoptotic effect, in particular on HeLa cells. The inhibition activity against RTK was identified as the most likely mechanism for the cytotoxic effect of these complexes. Compounds C1 and C2 exhibited a significant pro-apoptotic effect on HeLa cancer cells. Among the IC50 concentrations applied for 24 h, C1 had the greatest effect on increasing the proportion of HeLa cells in the subG1 phase in the cell cycle (20.93% ± 4.96%, 4.55% ± 0.65% compared to control). Similarly, compound C2 also significantly increased the proportion of HeLa cells in the subG1 phase of the cell cycle, resulting in a decreased number of cells in G1 (Avdović et al., 2022). Figure 7 shows coumarin–palladium (II) complex effects on cellular processes, including cell cycle regulation, DNA damage repair, COX-2, and PI3K.

By heating 7-hydroxy-4-methylcoumarin with acetic anhydride and anhydrous alumina in methanesulfonic acid to synthesize the compound 6-acetyl-7-hydroxy-4-methylcoumarin, Hejchman et al. developed a new coumarin derivative using microwave assistance and achieved a yield of 40% (Hejchman et al., 2019). Two series of Schiff bases (8–14 and 18–28) were synthesized, consisting of combinations of salicylaldehyde or 7-hydroxycoumarin backbones and para-substituted aniline. This research evaluated the cytotoxic activity of Schiff bases against tumor cell lines, specifically CFPAC-1 and HeLa cells. The efficacy of the compounds was determined on the basis of their IC50 values, which are the concentrations required for 50% inhibition of cell growth. The study also calculated the selectivity index to measure the ability of the compounds to target tumor cells, while minimizing damage to normal cells (Emami and Dadashpour, 2015). Compounds 8–14 and 18–28 were evaluated for their antitumor activity and selectivity across CFPAC-1 and HeLa tumor cell lines, as well as NIH-3T3 fibroblasts. Compounds were classified according to their cytotoxic activity as ineffective (IC50 values from 499 to 100 µM), less effective (IC50 between 11 and 99 µM), and effective (IC50 ≤ 10 µM). Most of the synthesized Schiff bases showed moderate activity against tumor cells, falling into the “less effective” category. It is worth noting that Compound 20 was highly effective against CFPAC-1 cells, while Compounds 8 and 9 were ineffective against both tumor cell lines. Compound 22 exhibited resistance in HeLa cells but limited sensitivity in CFPAC-1 cells. However, compared to known chemotherapeutic agents working at nanomolar concentrations, the tested compounds showed significantly lower cytotoxicity. Compound 14 was effective against NIH3T3 cells with an IC50 of 4 μM, whereas compound 20 was effective against CFPAC-1 cells with an IC50 of 10 μ (Hejchman et al., 2019).

Ma et al investigated the effects of a new microtubule-targeting agent, 6-chloro-4-(methoxyphenyl) coumarin (CMC), on HeLa cells. The ability of CMC to arrest the cell cycle at the G2-M phase and trigger apoptosis in HeLa cells was explored. CMC exhibited potent anticancer activity on various human cancer cell lines. Tests conducted on nine different cancer cell lines demonstrated that CMC is an effective anticancer agent. Among the observed coumarin analogs, CMC exhibited the best anticancer activity. According to the results of the MTT test on HeLa cells, the IC50 value of CMC was determined to be 0.63 μmol/L. CMC induced G2-M phase arrest and increased apoptosis in HeLa cells. CMC was effective in HeLa cells through microtubule depolymerization and caused arrest in the cell cycle at the G2-M phase. It has been determined that CMC reversibly induces G2-M phase arrest and increases apoptosis in a dose- and time-dependent manner (Ma et al., 2012).

In another study, the synthesis of thiomorpholine–coumarin derivatives induced apoptotic cell death and G1 phase arrest in the cell cycle. Coumarinated oxazole derivatives showed potent anticancer activity in HeLa cells (Ayati et al., 2018).

**FIGURE 8:**
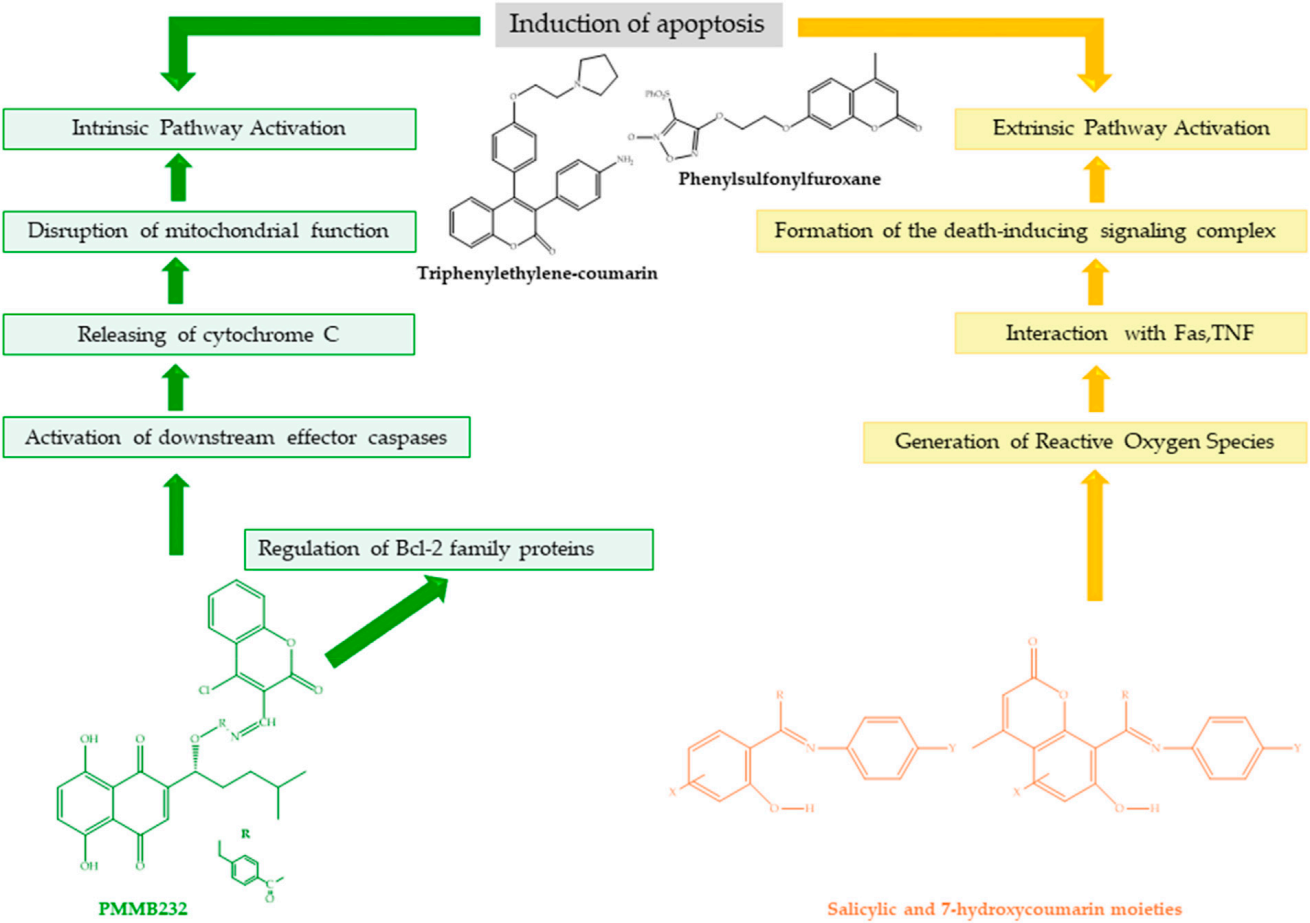
Structure and apoptotic mechanisms of salicylic and 7-hydroxycoumarin moieties, PMMB232, triphenylethylene–coumarin, and phenylsulfonylfuroxane.

Han et al. synthesized shikonin–coumarin carboxylic acid derivatives and evaluated their antihuman cervical cancer activity. The coumarin derivative PMMB232 showed high antiproliferative activity in human cervical cancer cells with an IC50 value of 3.25 ± 0.35 µM. The mechanism of action of PMMB232 was investigated for its role in inducing apoptosis in cancer cells by examining the expressions of HIF-1 and E-cadherin in HeLa cells. The study discovered that the expression of HIF-1 decreased, while the expression of the E-cadherin protein increased. Additionally, PDK1 protein levels were found to be lower in HeLa cells, while PDH-E1 expression increased. In the interaction model, PMMB232 is tightly bound to the HIF-1α-binding site through four hydrogen bonds with His 115. The mitochondrial membrane potential was reduced by PMMB232, resulting in apoptosis in human cervical cancer cells. Apoptosis can also be induced by the excessive production of reactive oxygen species (ROS) in cells. ROS has been used to eliminate cancer cells due to their ability to cause severe cell damage and death. Han et al. found that shikonin treatment significantly increased ROS production in human cervical cancer cells compared to controls. Researchers synthesized a shikonin derivative that disrupts glycolysis and promotes apoptosis in cancer cells. It also enhances cell adhesion and inhibited cancer cells from migrating and metastasizing (Han et al., 2018). Figure 8 illustrates the inhibition of apoptosis by the PMMB232 coumarin derivative through the BCL-2 protein family.

The coumarin–chalcone conjugate showed significant *in vitro* anticancer activity against C33A (cervical carcinoma) cells. The IC50 ranged from 3.59 to 81.10 µM (Sashidhara et al., 2010). Further investigations indicated that by inducing apoptosis and arresting the cell cycle in the G2/M phase, this hybrid inhibited the proliferation of cervical cancer cells (HeLa and C33A) (Singh et al., 2014). The apoptotic effect was mediated by the induction of the caspase-dependent intrinsic pathway and changes in cellular levels of Bcl-2 family proteins (Ashraf et al., 2017).

Makowska et al. conducted a study on two series of compounds: Series A-I (compounds 3–9) and Series A-II (compounds 10–16). The compounds in Series A-I are 3-(benzoxazol-2-yl)-2-iminocoumarins, while those in Series A-II are 3-(benzothiazol-2-yl)-2-iminocoumarins. The classification of these compounds is based on the heterocyclic substituents attached to the 2-iminocoumarin scaffold, which are either benzoxazole or benzothiazole. The *in vitro* cytotoxic activity of two hybrid series, series A-I (21.26a, a-d) and series A-II (21.26aʹ-21.26dʹ), was evaluated against the ovarian cancer cell line A427 and cervical cancer cell line SISO. The A-I series 21.26d was found to induce apoptosis in two representative cell lines and was impressively cytotoxic, with an IC50 below 0.01–0.30 µM against the A427 ovarian cancer cell line and the SISO cervical cancer cell line. SAR investigation revealed that the antiproliferative activity of 2-iminocoumarins is enhanced by the presence of a benzoxazole or benzothiazole ring system at position 3 (Makowska et al., 2019).

These studies critically compare the efficacy of coumarin-based compounds in inducing apoptosis in cancer cells and highlight various aspects of their anticancer properties. In HeLa cells in particular, these compounds were effective in arresting the cell cycle and inducing apoptosis. Researchers have explored various pathways by which coumarin derivatives exert their effects, such as microtubule depolymerization, modulation of apoptosis-related protein expression, and cell cycle regulation. Studies by Ma et al. (2012) and Ayati et al. (2018) have highlighted the potent anticancer activity of coumarin derivatives such as CMC and thiomorpholine–coumarin derivatives in various cancer cell lines. These compounds have shown dose- and time-dependent effects on cancer cell proliferation and viability, demonstrating their potential as effective anticancer agents. Moreover, Han et al. (2018) has investigated the mechanisms of action of coumarin derivatives, such as PMMB232, in inducing apoptosis in cervical cancer cells. These compounds were shown to affect key proteins involved in apoptosis, cell adhesion, and glycolysis, leading to disruption of the mitochondrial membrane potential and increased production of reactive oxygen species (ROS), ultimately resulting in cancer cell death. Makowska et al. (2019) have investigated the structure–activity relationship (SAR) of coumarin derivatives with heterocyclic substituents, demonstrating how specific structural modifications can enhance the antiproliferative activity of these compounds. The presence of benzoxazole or benzothiazole rings at specific positions in the coumarin scaffold has been found to significantly influence cytotoxicity against ovarian and cervical cancer cell lines. Furthermore, Avdović et al. (2022) synthesized novel coumarin–palladium (II) complexes and evaluated their cytotoxic effects on human cancer cells, including HeLa cells. These complexes exhibited pro-apoptotic effects and promising cytotoxic activity, possibly through receptor tyrosine kinase (RTK) inhibition, demonstrating their potential as effective anticancer agents. In conclusion, these studies collectively demonstrate the diverse mechanisms by which coumarin-based compounds exert their anticancer effects and highlight their potential as promising candidates for the development of novel anticancer therapies targeting various types of cancer.

Platinum-based chemotherapies (e.g., cisplatin and carboplatin) are commonly employed in the treatment of ovarian cancer. Nevertheless, patients who initially respond to treatment may develop resistance over time, which reduces the efficacy of the treatment. The development of platinum resistance is associated with a number of mechanisms, including the upregulation of DNA repair mechanisms, increased drug detoxification pathways, and decreased intracellular accumulation of drugs. The study was designed to investigate the effects of 4-hydroxycoumarin–platinum (IV) complexes on the SKOV-3 ovarian cancer cell line. The 4-hydroxycoumarin-platinum (II) complex has an effect similar to that of the standard drugs cisplatin and oxaliplatin. The study also demonstrated the potential of compounds derived from oxaliplatin to overcome resistance to cisplatin and to be effective against resistant cell lines. All targeted compounds, particularly oxoplatins B1 (IC50 ≤ 15.2 μM), B2, and acid A1 (IC50 ≤ 15.2 μM), showed significant activity. Compounds with various platinum cores and linkages were discovered to possess distinct antitumor properties. In particular, compounds derived from oxaliplatin exhibited resistance-breaking properties. They were also active against normal cells. These findings suggest that 4-hydroxycoumarin–platinum (IV) complexes may have much potential in the treatment of ovarian cancer. The reduction process is involved in the mechanism of the anticancer activity of 4-hydroxycoumarin–platinum (IV) complexes. Reducing agents interact with these complexes to yield platinum (II) complexes. The resultant platinum (II) complexes have DNA interactions and DNA damage. The prodrug of the platinum (IV) complex of 4-hydroxycoumarin is reduced to form the platinum (IV) complex (Li et al., 2019). DNA damage and reduction potential of platinum (IV) compounds and 4-hydroxycoumarin–platinum (II) compounds are shown in Figure 7.

Meng et al. prepared eight new platinum (II) complexes (Pt1–Pt8) containing substituted 3-(2′-benzimidazolyl)-coumarins and evaluated their cytotoxic activity against cisplatin-resistant SKOV-3/DDP cancer cells. In this study, Pt1–Pt8 exhibited higher *in vitro* cytotoxicity against human SKOV-3/DDP tumor cells than cisplatin, with IC50 values ranging from 1.01 to 10.32 μM. Among them, Pt1–Pt8 showed the highest level of sensitivity to the SKOV-3/DDP cell line. However, Pt1–Pt8 were also slightly cytotoxic to normal HL-7702 cells. Additionally, Pt1 (1.0 μM) induced apoptosis in approximately 43.0% of SKOV-3/DDP tumor cells. Compared to cells treated with cisplatin (70.0 μM), Pt2 (3.0 μM), and Pt3 (10.0 μM), only 8.2%, 19.1%, and 11.2%, respectively, underwent apoptosis, while using Pt1 (1.0 μM) resulted in a significantly higher proportion of apoptotic cells. The induction of Pt1–Pt3 targeted mitochondrial dysfunction signaling pathways, leading to apoptosis in SKOV-3/DDP cancer cells. The telomerase inhibitor Pt1, which targets c-Myc promoter elements, was highly effective in inhibiting telomerase activity, with an inhibition rate of 52.28%. Pt2 (3.0 μM), Pt3 (10.0 μM), Pt4 (3.0 μM), and Pt5 (10.0 μM) had inhibition rates of 39.57%, 35.90%, 14.55%, and 11.82%, respectively (Meng et al., 2018).

Two new platinum (II) complexes, [PtCl2(H-MeOBC) (DMSO)] (Pt1) and [Pt2Cl3 (MeOBC) (DMSO)2] (Pt2), were synthesized with 3-(20)-benzimidazolyl)-8-methoxycoumarin (H-MeOBC) as ligands, and their antiproliferative activity against SKOV-3 and HeLa cancer cells was determined by Qin et al. The MTT assay was used to evaluate Pt1 and Pt2 activity *in vitro* against human HeLa, cisplatin-resistant SKOV-3/DDP, and SKOV-3 cancer cells, with cisplatin as a positive control. Pt1 and Pt2 exhibited potent cytotoxicity against SKOV-3/DDP cancer cells with IC50 values of 10.3 ± 0.3 mM and 0.5 ± 0.2 mM, respectively. The synthesized Pt (II) complexes showed enhanced cytotoxicity against the tested SKOV-3/DDP cells compared to cisplatin (cisplatin IC50 = 70.3 ± 1.9 mM). It is important to note that Pt1 and Pt2 showed low cytotoxicity against normal cells. The selectivity of Pt1 and Pt2 for the SKOV-3/DDP cancer cell line is indicated by their effect on HL-7702 cells. Telomerase expression was significantly inhibited in Pt2-treated cells (56.57% inhibition) compared to Pt1-treated cells (37.07% inhibition). Pt2, a double Pt (II) complex, causes cell cycle arrest at G2/M, apoptosis, and mitochondrial dysfunction (Qin et al., 2018).

Qin et al. (2018) and Meng et al. (2018) examined the cytotoxic activity of platinum (II) complexes against cisplatin-resistant SKOV-3/DDP cancer cells. Qin et al. (2018) synthesized Pt1 and Pt2 complexes with H-MeOBC ligands, which demonstrated potent cytotoxicity against SKOV-3/DDP cells, with IC50 values of 10.3 ± 0.3 μM and 0.5 ± 0.2 μM, respectively. The complexes exhibited enhanced cytotoxicity compared to cisplatin. The Pt2 complex was observed to induce cell cycle arrest at the G2/M phase, apoptosis, and mitochondrial dysfunction, accompanied by a significant inhibition of telomerase activity. Conversely, Meng et al. (2018) prepared Pt1–Pt8 complexes containing substituted 3-(2′-benzimidazolyl)-coumarins, which demonstrated higher cytotoxicity against SKOV-3/DDP cells than cisplatin, with IC50 values ranging from 1.01 to 10.32 μM. The complexes, designated as Pt1–Pt8, were observed to induce apoptosis in SKOV-3/DDP cells, with Pt1 exhibiting the highest sensitivity. Furthermore, these complexes were observed to target mitochondrial dysfunction pathways, resulting in apoptosis in cancer cells. In particular, Pt1 demonstrated a high degree of efficacy in inhibiting telomerase activity. In contrast, the study by Li et al. (2019) concentrated on 4-hydroxycoumarin–platinum (IV) complexes and their potential for the treatment of ovarian cancer. The oxaliplatin-derived complexes exhibited significant antitumor properties, including resistance-breaking capabilities against cisplatin-resistant cell lines. The reduction process of these complexes to platinum (II) forms was identified as a crucial mechanism for DNA interactions and damage, contributing to their anticancer effects. In summary, while Qin et al. (2018) and Meng et al. (2018) investigated the cytotoxicity and apoptotic effects of platinum (II) complexes against cisplatin-resistant cancer cells, Li et al. (2019) focused on the distinctive properties of 4-hydroxycoumarin–platinum (IV) complexes derived from oxaliplatin, emphasizing their potential to overcome resistance and induce DNA damage for the effective treatment of ovarian cancer. Further research integrating the findings of these studies could facilitate the development of more efficacious and targeted therapies for ovarian cancer. The development of resistance to drugs in cancer cells can significantly reduce the efficacy of treatment. To address this issue, studies such as those investigating 4-hydroxycoumarin–platinum (IV) complexes suggest the development of new compounds that may be effective against cell lines resistant to existing drugs.

The modulation of the major signaling pathways involved in platinum resistance by coumarin compounds has been shown to improve the efficacy of treatment regimens for gynecological cancers. Coumarin derivatives may have potential therapeutic effects in overcoming platinum resistance by modulating major signaling pathways involved in platinum resistance. For example, 4-hydroxycoumarin–platinum (IV) complexes and O-prenylated coumarin derivatives may be effective by modulating specific signaling pathways in cells that develop platinum resistance. It is believed that these derivatives may enhance the efficacy of platinum in cancer cells by targeting mechanisms associated with platinum resistance. Consequently, coumarin derivatives are a potential therapeutic strategy by targeting signaling pathways linked to platinum resistance.

Thiazoles are heterocyclic compounds containing both nitrogen and sulfur. They have various pharmacological activities and can be found in nature or synthesized. Among the thiazoles, coumarin is an effective anticancer agent. As an anticancer drug scaffold, coumarin with a triazole moiety is used (Ibrar et al., 2016). The triazole moiety enhances the solubility and receptor/enzyme binding affinity (Zhao et al., 2019). Rawat and Reddy have studied the chemical structures of a number of coumarin derivatives based on the triazole moiety. To evaluate their cytotoxic activity against HeLa cancer cell lines, they designed fluorescent probes using triazolyl coumarin. The probes showed significant inhibition in HeLa cells, surpassing DOX (Rawat and Reddy, 2022). Coumarin–benzimidazole hybrids have shown potential against ovarian cancer cells (SKOV-3 and OVCAR-8) in the concentration range of 0.07–3.57 µM (Liu et al., 2015). Significant anticancer activity was demonstrated by coumarin–benzothiazole and coumarin–thiadiazole hybrids. At concentrations of 0.24 and 0.33 µM, the coumarin–benzothiazole compound showed activity against ovarian cancer cell (OVCAR-4) lines (Gabr, 2018; Mah et al., 2019). A study found that imine-linked coumarin–thiazole hybrids (IC50 = 0.0091–0.5689 µM) had significant antiproliferative activity against HeLa cells and were more potent than doxorubicin (IC50 = 1.1073 µM) (Abd El-Karim et al., 2019).

The reaction between 4-chloromethylenecoumarin and *N-*benzyl benzimidazole derivatives afforded coumarin-substituted benzimidazolium chlorides and 6-substituted 4-chloromethylenecoumarin derivatives. Karataş et al reacted 4-chloromethylene coumarin with *N-*benzyl benzimidazole derivatives, and the three variations of benzimidazolium chloride (compounds 21.24 (a–c)) were synthesized to test their anticancer activity. The cytotoxicity of the hybrids was tested on A2780 ovarian cancer cells at a concentration of 100 μM. All compounds showed significant cytotoxicity, with the simple benzyl-substituted salts being more cytotoxic than the other compounds against the A2780 cell line. On the other hand, the methylated derivatives showed greater activity against A2780 at 100 μM than all the coumarin salts (Karataş et al., 2019). All of the compounds in the study had lower cytotoxicity than docetaxel, which may cause side effects in clinical use (Sato et al., 2013).

Six fluorescent half-sandwich iridium (III) coumarin–salicylaldehyde Schiff base compounds have been synthesized and characterized by Liu et al. The introduction of the coumarin unit resulted in an increase in the antitumor activity. The best compound exhibited antitumor activity almost twice that of clinical cisplatin. The study demonstrated that the compounds interfered with the cellular uptake mechanism, leading to their accumulation in lysosomes. This, in turn, disrupted the integrity of lysosomes and induced apoptosis. Compounds Ir-1 to Ir-6 show antitumor activity with IC50 values ranging from 9.9 ± 0.1 µM to 40.7 ± 12.9 µM when compared to the coumarin-free Ir-3 semi-sandwich (Ir-7). Under the same conditions, the best compound, Ir-2, almost doubled the activity of cisplatin (widely used in clinical studies). These results indicate that the antitumor activity of Ir-3 compounds may be enhanced by the introduction of coumarin. Furthermore, the best compound exhibited almost twice the antitumor activity of clinical cisplatin (Liu et al., 2020).

Maleki et al. evaluated the cytotoxic and anticancer properties of *o*-prenylated coumarin derivatives on cervical cancer HeLa cells. Cisplatin was used as a positive control to compare the cytotoxic effects of O-prenylated coumarin derivatives with those of drugs commonly used in the clinic. The study showed that twelve *o*-prenylated coumarin derivatives are toxic to HeLa cancer cells and that certain *o*-prenylated coumarin derivatives can inhibit the growth of HeLa cells by inducing apoptosis. Upon exposure to these coumarins, HeLa cells underwent G1 cell cycle arrest and reduced S-phase (Maleki et al., 2020).

Imine is a versatile pharmacophore present in numerous drugs, including cefixime and gemifloxacin. It has the potential to perform various noncovalent interactions, such as hydrogen bonds and metal coordination, with active sites in organisms. Imine also plays a vital role in designing new drugs (Motaleb and Selim, 2019).

Kumar et al. showed that thiazole-linked coumarin–imine hybrids and coumarinyl-linked pyrazolylthiazole hybrids were highly antiproliferative against the HeLa cancer cell line, with IC50 values ranging from 1.29 to 50 µM (Kumar et al., 2018).

Metal complexes play a crucial role in biochemical processes, as they accelerate the action of the drug and allow for a better delivery of the drug. The pharmacological activity of coumarin-conjugated metals is largely dependent on the nature of the coordinating metal (Keri et al., 2015). The authors have designed and synthesized Ir (III) complexes linked to coumarin as theranostic anticancer agents capable of overcoming the resistance of cisplatin. Each derivative was found to be potent and prevented mitochondrial dysfunction and increased ROS in HeLa cells (Ye et al., 2015).

The coumarin pyrazole hybrids (IC50 = 7–148 µM) and analogs (IC50 = 59–123 µM) showed a wide range of activities against HeLa but were less potent than doxorubicin (IC50 = 2.5–8 µM) (Vaarla et al., 2015; Vaarla et al., 2019).

Several studies have synthesized a hybrid of coumarin and phenylsulfonylfuroxane, which has been found to exhibit an antiproliferative effect on various cancer cells. The IC50 values of coumarin and phenylsulfonylfuroxane for solid cervical and ovarian cancer cell lines (HeLa, SKOV-3, OVCA429, OVCA433, and A2780) ranged from 20.9 to 445 nM in experiments. For cisplatin-resistant A2780/CDDP cell lines, the values ranged from 24.9 to 156.8 nM. The studies demonstrated that the compound induced apoptosis and arrested the cell cycle in the G2/M phase of the A2780 cell line, providing evidence for its anticancer mechanism of action. Additionally, the compound interferes with the phosphorylation of MEK1 and ERK1 (Liu et al., 2014; Wang et al., 2018; Guo et al., 2018).

The coumarin–furoxan hybrid showed significant activity against HeLa, with the majority of the compounds exhibiting more potent activity than doxorubicin (IC50 = 10.21 µM) (Zhang et al., 2018). The apoptotic effect of the phenylsulfonyl-furoxane compound is given in Figure 8.

Among the studies reviewed, coumarin derivatives and thiazoles appear to have potential anticancer effects against ovarian cancer cells by different mechanisms. For example, coumarin–benzimidazole hybrids and coumarin–thiadiazole hybrids exhibit significant anticancer activity even at low concentrations, while coumarin–furoxane hybrids and coumarin-imine hybrids can induce apoptosis by arresting the cell cycle. It has been demonstrated that coumarin compounds linked with metal complexes may be effective against resistant cells and prevent mitochondrial dysfunction. Conversely, iridium compounds containing coumarin may affect the intracellular uptake mechanism and trigger apoptosis by increasing their antitumor activity. These compounds have been shown to almost double the activity of cisplatin, which is widely used in the clinic. Furthermore, it has been demonstrated that the solubility of coumarin derivatives coupled with a triazole group is increased, and their receptor/enzyme binding affinity is enhanced. These studies indicate that different coumarin derivatives and thiazoles have various potentials in the treatment of ovarian cancer. It is emphasized that each compound acts on cancer cells by different mechanisms and may provide advantages against resistant cells.

It has been shown that Src kinase plays a crucial role in the regulation of the invasion and metastasis of cancer cells. Cinnamoylchromen-2-one and pyranochromen-2-one derivatives were investigated for their inhibitory effects on Src kinase and antiproliferative activity against SKOV-3 ovarian adenocarcinoma cells. The results indicated that cinnamoyl-2-one had a higher antiproliferative activity than pyranochromen-2-one. Cinnamoyl-2-one is highly toxic to ovarian cancer cells, inhibiting growth by 63% at a concentration of 50 µM. With an IC50 value of 14.5 µM, the compound also showed high inhibition of Src kinase. The cinnamoylchromen moiety at the coumarin position is more effective in inhibiting Src kinase (Chand et al., 2013).

El-Gamal and Oh synthesized a series of condensed tricyclic coumarin sulfonate derivatives (6-oxo-6,7,8,9,10,11-hexahydrocyclohepta [c]chromen-3-yl benzenesulfonate (1e), 6-Oxo-6,7,8,9,10,11-hexahydrocyclohepta [c]chromen-3-yl 4-methyl benzenesulfonate (1f), 6-oxo-6,7,8,9,10,11-hexahydrocyclohepta [c]chromen-3-yl 4-(trifluoromethyl) ben-zenesulfonate (1g) 6-oxo-6,7,8,9,10,11-hexahydrocyclohepta [c]chromen-3-yl 4-(tert-butyl) benzenesulfonate (1h), and 6-oxo-6,7,8,9,10,11-hexahydrocyclohepta [c]chromen-3-yl 4-fluoro-benzenesulfonate (1i)) and evaluated their cytotoxic activity against OVCAR-4 ovarian cancer cells. It was found that aromatic sulfonate derivatives 1e-i, 1-n (6-oxo-7,8,9,10,11,12-hexahydro-6H-cycloocta [c]chromen-3-yl benzenesulfonate), and 1-o (6-oxo-7,8,9,10,11,12-hexahydro-6H-cycloocta [c]chromen-3-yl methyl benzenesulfonate) had stronger antiproliferative activities than aliphatic and cyclopropyl analogs. The IC50 values for the OVCAR-4 cell line were found to be 8.13 µM for sulfonate derivatives 1h and 5.76 µM for sulfonate derivative 1i (El-Gamal and Oh, 2014).

A relationship exists between the sulfonate derivatives developed by El-Gamal and Oh (2014) and the Src kinase inhibitors studied by Chand et al. (2013). This indicates the potential of coumarin derivatives to exert a broad-spectrum effect against cancer cells. Both studies aim to extend the anticancer effects of coumarin derivatives through different targets. The objective of these studies is to gain a deeper understanding of the molecular processes targeted in cancer therapy and to develop more effective therapies against resistant cancer types. Coumarin derivatives have emerged as promising candidates in cancer therapy, offering a broad spectrum of action and targeting various cell pathways. The development and diversification of these compounds provide in-depth information on cancer biology, expanding treatment options and opening the doors to more effective treatment methods.

To synthesize derivatives of 3-(coumarin-3-yl)-acrolein and investigate their anticancer activities, Chen and colleagues conducted a study. The study synthesized two series of hybrids, 5a-g, and 6a-g, containing 3-acetylcoumarin derivatives and 3-(coumarin-3-yl)-acrolein derivatives, respectively. The research showed that most of the synthesized compounds exhibited potent inhibitory activity against cancer cells while exhibiting low cytotoxicity to normal cells. Novel hybrids of the 5a-g and 6a-g series were designed and synthesized in this study. HeLa cancer cell lines and HUVEC and LO2 normal cell lines were used to evaluate the antiproliferative activities of compounds 5d and 6e. Results indicated that compounds 5d and 6e were most effective against tested cancer cell lines. Compound 5d exhibited IC50 values ranging from 0.70 ± 0.05 to 4.23 ± 0.15 μM, while compound 6e exhibited IC50 values ranging from 0.39 ± 0.07 to 14.82 ± 0.28 μM. Compounds 5d and 6e were found to be active against several cancer cell lines. Compound 6e was found to suppress cell migration, inhibit invasion, and enhance apoptosis. Mechanistic studies have also shown that it inhibits the PI3K/AKT signaling pathway, which leads to mitochondria-dependent apoptosis. The study highlights the potential of 3-(coumarin-3-yl)-acrolein derivatives as novel anticancer agents and represents an important step toward their clinical development (Chen et al., 2023). Chen et al. (2023) reported that 3-(coumarin-3-yl)-acrolein derivatives exhibited strong inhibitory activity against cancer cells and low cytotoxicity to normal cells. In particular, it was emphasized that compounds 5d and 6e were effective against various cancer cell lines, with 6e demonstrating the ability to suppress cell migration, inhibit invasion, and increase apoptosis.

In a study by Koparde et al., coumarin–maltol hybrids (2a–j) were synthesized, characterized, and evaluated for *in vitro* activity against human cancer cell lines. In addition, they carried out studies on the molecular binding and DNA cleavage of these compounds. The results suggest that these microwave-assisted synthesized compounds could be effective in treating cancer and microbial infections. Compounds (2a) and (2d) showed strong cytotoxicity against HeLa cell lines. The IC50 values ranged from 2.47 to 4.26 μM. With IC50 values ranging from 2.47 to 9.21 μM, substitutions of methyl, methoxy, and tert-butyl at the C-6 position of the coumarin moiety showed excellent-to-moderate activity. IC50 values ranging from 2.47 to 14.64 μM were observed for compounds (2a), (2d), (2g), (2i), (2j), and (2k) against the A549 cell line. Two compounds were the most active. Compounds (2a), (2d), (2g), and (2j) showed IC50 values from 3.98 to 10.16 μM against the HeLa cell line, among which (2a) was the most cytotoxic with IC50 of 3.98 μM. DNA cleavage studies were performed to investigate the mechanism of action of the synthesized coumarin–maltol hybrids against cancer. Compounds (2a), (2j), and (2k) caused complete DNA cleavage, producing low-molecular-weight fragments that drifted on the gel during electrophoresis. The findings suggest that the substances could have a mechanism of action that inhibits the growth of cancer-causing organisms (Koparde et al., 2018). It has been reported that coumarin–maltol hybrids synthesized by Koparde et al. are effective against human cancer cell lines and can induce DNA double-strand breakage. It was reported that these compounds exhibited strong cytotoxicity, particularly against HeLa cell lines. Furthermore, different substituents were found to have a significant effect on the activity of the compounds.

Lu et al. synthesized 20 new acylhydrazone and 4-chlorocoumarin-substituted 1,5-diarylpyrazole benzenesulfonamide derivatives designated as compounds 7a–7t. These compounds are acylhydrazone and 4-chlorocoumarin-substituted 1,5-diarylpyrazoles. These compounds have been designed and tested for their efficacy in the inhibition of COX-2 and the suppression of cancer proliferation and metastasis. Compound 7t exhibited significant selective COX-2 inhibition and potent anticancer activity. This made it a standout candidate in the study. The synthesized coumarin–sulfonamide hybrids showed potential activity against the HeLa cancer cell line (IC50 = 0.36–40.14 µM) and low cytotoxicity against normal 293 T and L02 cells (IC50 = 101.24->300 µM). Lu et al. showed that treatment effectively induced dose- and time-dependent apoptosis in HeLa cells. It also significantly inhibited cancer cells from adhering, migrating, and invading, thereby preventing metastasis. Compound 7t was identified as a potent COX-2 inhibitor with significant anticancer properties in the study of coumarin sulfonamide derivatives. Compound 7t exhibited potent inhibitory and antiproliferative effects. It induced apoptosis in cancer cells and suppressed metastasis-related processes. Docking simulations supported its binding affinity to COX-2, suggesting its potential as a selective inhibitor. The antiproliferative activities of the compounds were enhanced by the inclusion of specific moieties, while toxicity was reduced (Lu et al., 2016). Lu et al. (2016) reported that coumarin–sulfonamide hybrids demonstrated potential anticancer activity against the HeLa cancer cell line and exhibited low cytotoxicity to normal cells. In particular, it is emphasized that compound 7t is effective as a COX-2 inhibitor and induces apoptosis in cancer cells.

The study by Chen et al. (2023) shows that 3-(coumarin-3-yl)-acrolein derivatives are effective against a wide range of cancer cells. In contrast, the study by Koparde et al. (2018) indicates that coumarin–maltol hybrids show strong cytotoxicity, especially against HeLa cells. Lu et al. (2016) emphasized that coumarin–sulfonamide hybrids are effective as COX-2 inhibitors and induce apoptosis in cancer cells. Chen et al. (2023) indicated that 3-(coumarin-3-yl)-acrolein derivatives may lead to mitochondria-dependent apoptosis by inhibiting the PI3K/AKT signaling pathway. Koparde et al. (2018) reported that coumarin–maltol hybrids can induce DNA double-strand breaks in cancer cells. Lu et al. (2016) demonstrated that coumarin–sulfonamide hybrids, which act as COX-2 inhibitors, induce apoptosis in cancer cells and suppress metastasis. This assessment highlights the similarities and differences between studies evaluating the potential efficacy of different coumarin derivatives against cancer cells. Each study provides important findings by targeting cancer cells through different mechanisms.

The PI3K signaling pathway plays a crucial role in tumorigenesis and other cellular processes. Because of its role in regulating cell growth, survival, and metastasis, targeting key molecules in this pathway is an important alternative approach to cancer therapy (Mayer and Arteaga, 2016; O’Donnell et al., 2018). Wang et al. synthesized a series of new coumarin–benzylsulfone derivatives and tested their enzymatic activities against PI3Ks using ELISA with rigosertib as a positive control. Compound 5 is a series of benzylsulfone coumarin derivatives, which are designated as compounds 5a to 5o. The compounds have been synthesized and characterized concerning their kinase inhibitory activity and their antitumor potential. Each of the compounds was evaluated for their cytotoxicity against a variety of cancer cell lines, including HeLa. Compound 5h showed the most potent activity against these cell lines. Its IC50 values ranged from 18.12 to 32.60 μM. Coumarin–benzylsulfone derivatives exhibited an inhibition rate of 50.8%, while rigosertib exhibited a rate of 53.2% at a concentration of 20 µM when evaluated as PI3K inhibitors. The benzylsulfone coumarin derivatives in this study showed significant kinase inhibitory activity. In particular, they inhibited PI3K. Indicating their potential as anticancer agents, most of the synthesized compounds showed significant activity against PI3K. Among the tested compounds, 5h exhibited the most potent activity against a panel of tumor cell lines with IC50 values between 18.12 and 32.60 μM, followed by 5 m with IC50 values between 29.30 and 42.14 μM. In addition, *in silico* molecular docking studies indicated that compounds 5h and 5m could be promising PI3K inhibitors, suggesting their potential for further investigation in the treatment of cancer (Wang et al., 2019a).

In cervical cancer cell lines such as HeLa, HT-3, and CaSki, protein expression of p38 mitogen-activated protein kinase (MAPK) has been demonstrated. This suggests an important role for p38 MAPK in the development of cervical cancer. MAPK pathway inhibition has been proposed as a potential cervical cancer treatment. Studies have shown that inhibition of p38 MAPK phosphorylation disrupts the nuclear translocation of NF-kB. This leads to the suppression of migration and the invasion of human cervical cancer cells. Batran et al. designed and synthesized a series of coumarinated hybrid molecules to evaluate their activity against cervical cancer. At concentrations of 10 µM (80%) and 30 µM (72%), the thioxopyrimidine compound showed a significant decrease in cell viability against HeLa cells. Moreover, the synthesized thioxopyrimidine derivative showed inhibition of p38 MAPK phosphorylation (p38 MAPK activation = 0.64 ± 0.2) (Batran et al., 2017).

This study proposes that targeting the p38 MAPK pathway may be a potential strategy in the treatment of cervical cancer. Conversely, benzylsulfone coumarin derivatives synthesized by Wang et al. (2019) demonstrated antitumor activity by targeting the PI3K signaling pathway. This highlights the potential of the PI3K signaling pathway as a valuable alternative in cancer treatment. The human papillomavirus (HPV) is the primary causative agent of cervical cancer. The oncogenic processes induced by HPV can result in cancerous cells by disrupting the mechanisms that regulate the cell cycle and inhibiting apoptosis. The coumarin derivatives developed by Batran et al. (2017) demonstrated effects via the p38 MAPK pathway in cervical cancer cell lines. This study demonstrates the potential for the inhibition of cancer cell growth by the targeting of signaling pathways activated by HPV infection.

Hao et al. synthesized conjugates of podophyllotoxin and coumarin and investigated their cytotoxicity on HeLa and LoVo cells. With IC50 values of 9.7 ± 1.8 μM in HeLa cells and 4.9 ± 0.6 μM in LoVo cells, podophyllotoxin–coumarin derivatives exhibited the most potent cytotoxic effects. Furthermore, 14e was found to disrupt microtubule dynamics and regulate P21 and cyclin D1 in LoVo cells by arresting the cell cycle in the G1 phase. These results suggest that 14e is potently cytotoxic to the cancer cell lines tested (Hao et al., 2019).

Ma and Liu synthesized coumarin derivatives to develop a new anticancer agent that targets the PI3K/Akt signaling pathway. Coumarin derivatives containing pyridinyl urea units were numbered as 4a-m (4a: 2,5-dimethoxyphenyl derivative; 4b: 4-fluorophenyl derivative; 4c: 4-chlorophenyl derivative; 4d: 4-methoxyphenyl derivative; 4e: 4-methylphenyl derivative; 4f: 4-hydroxyphenyl derivative; 4g: 2,5-dimethoxyphenyl derivative; 4h: 3-chlorophenyl derivative; 4i: 4-fluorophenyl derivative; 4j: 4-methyl-3-acetylphenyl derivative; 4k: 3,4-difluorophenyl derivative; 4l: 3,4-dimethylphenyl derivative; 4 m: 3-chloro-4-fluorophenyl derivative) in the study. The study evaluated the inhibitory effects of these derivatives on tumor cell growth using the MTT assay. Some of the coumarin derivatives examined in the study were more sensitive to HeLa cells and showed better or comparable efficacy. The growth of HeLa cells was inhibited by compounds 4e, 4g, and 4h, which had IC50 values of 2.27, 2.39, and 2.17 μM, respectively. Compound 4h was found to be almost twice as effective as −511 (IC50 = 4.20 μM). The study investigated the growth-inhibiting activities of coumarin derivatives in tumor cells and PI3K isoform selectivity. Coumarin analogs have been demonstrated to significantly inhibit the growth of HeLa cell lines. More potent PI3K inhibitors than existing compounds were identified for certain analogs. Specifically, compounds 4b and 4h were found to be much more effective PI3K inhibitors than the others. Furthermore, compound 4h was found to induce apoptosis by suppressing Akt phosphorylation in K562 cells and was identified as a selective PI3Kα/β/δ inhibitor. In general, the derivatives demonstrated higher sensitivity to HeLa cells and selectivity toward certain PI3K isoforms. The results indicate that the designed coumarin derivatives have the potential to act as novel PI3K inhibitors with anticancer properties (Ma and Liu, 2017).

Triphenylethylene–coumarin hybrids with amide side chains were synthesized by Tan et al. The malonic acid-linked dimers showed high antiproliferative activity against the HeLa cell line, with approximate IC50 values of 10 μM. By inhibiting tumor cell growth while sparing normal cells, triphenylethylene–coumarin hybrids exhibit a selective anticancer mechanism. These compounds have potent antiproliferative effects through interaction with DNA, which can disrupt processes vital for cancer cell survival. The efficacy of these compounds in inhibiting cell proliferation and binding to DNA is significantly influenced by the structure–activity relationships, particularly the linker length. The anticancer activity of triphenylethylene–coumarin hybrids involves targeting cancer cell growth, interacting with DNA, and optimizing structure–activity relationships to enhance their efficacy as potential anticancer agents (Tan et al., 2014). Figure 8 shows that triphenylethylene–coumarin inhibits cancer cell proliferation and DNA binding.

The diethylene glycol-bound isatin–coumarin hybrids showed weak-to-moderate anticancer activity against several cancer cell lines, including HeLa and SKOV-3, when bound to diethylene glycol with an IC50 range of 10.28- > 50 µM. It was more effective against HeLa than etoposide (with an IC50 > 50 µM). The analysis of the structure–activity relationship (SAR) showed that the anticancer activity was significantly affected by substituents on the C-3 and C-5 positions of the isatin motif. Electron-withdrawing groups such as -Cl and -F at the C-5 position were preferred. Electron-donating groups such as -Me were not favored. According to Fan et al., isatin–coumarin hybrids have the potential to combat cancer cell growth through various pathways. They can inhibit cancer cell proliferation and induce programmed cell death (apoptosis) by targeting specific molecular pathways essential for cancer cell survival. These compounds disrupt key processes that drive tumor growth. They can also cause DNA damage in cancer cells, leading to cell cycle arrest or cell death. Isatin–coumarin hybrids may have anti-angiogenic effects. They inhibit the formation of new blood vessels that feed tumors, thereby limiting their nutrient supply and inhibiting their growth (Fan et al., 2018).

The studies by Tan et al. (2014) and Fan et al. (2018) investigated the antiproliferative effects of different coumarin derivatives against cancer cells. It was reported that triphenylethylene–coumarin hybrids inhibit the growth of cancer cells by interacting with DNA, with the nature of this interaction being significantly affected by structure–activity relationships. Similarly, isatin–coumarin hybrids were also found to target cancer cell growth, with structural–activity relationships significantly affecting this activity.

Benci et al. conducted a study to evaluate the cytostatic activities of novel coumarin derivatives. The syntheses contain different heterocyclic groups, including 1,2,4-triazole derivatives (compounds 3–6), 4,5-dicianoimidazole derivative (compound 7), purine derivatives (compounds 8–13), and an acyclic nucleoside analog (compound 18). Human tumor cell lines and normal human fibroblasts were used to test the cytostatic activity of the synthesized compounds. Compound 6 and compound 10 showed moderate cytostatic activity against the HeLa cell line, with IC50 values of 35 μM and 33 μM, respectively, based on MTT assay data. The syntheses showed moderate antiproliferative effects on the tested cell lines without cytotoxicity in normal human fibroblasts. Only compounds 6 and 10 showed stronger effects. The study does not specify the anticancer mechanisms of action of the synthesized compounds. However, possible mechanisms include 1,2,4-triazole compounds affecting cellular signaling pathways, purine compounds affecting cellular metabolism, and acyclic nucleoside analogs that inhibit nucleic acid synthesis (Benci et al., 2012). Figure 7 illustrates the inhibition of EGF and VEGF by coumarin derivatives containing 1,2,4-triazole derivatives.

The study by Benci et al. (2012) demonstrates that 1,2,4-triazole and purine derivatives can influence cell growth and selectively target tumor cells without damaging normal cells. This underscores the synthesis of prospective novel compounds that target distinct mechanisms and selectively inhibit cancer cells. In contrast, studies by Tan et al. (2014), Batran et al. (2017), Ma and Liu (2017), Fan et al. (2018), Hao et al. (2019), and Wang et al. (2019), have focused on coumarin derivatives that target specific signaling pathways. For instance, studies that have focused on specific targets, such as p38 MAPK and PI3K signaling pathways, have demonstrated antitumor effects by affecting specific mechanisms in cancer cells. The *in vitro* study by Benci et al. (2012) encompassed a diverse array of compounds, whereas other studies concentrated on more narrowly defined targets. This discrepancy underscores the necessity to achieve a balance between the development of multi-targeted compounds that offer a comprehensive range of effects for cancer therapy and more specific compounds that target specific signaling pathways. The integration of these studies may facilitate the advancement of more efficacious and selective cancer therapies in future research.

Coumarin derivatives are currently being investigated as potential therapeutic agents for the treatment of gynecological cancers, with a particular focus on targeting specific mutations associated with these cancers. Singh et al. (2019) stated that cancer depends on multiple receptors or signaling pathways and emphasized the importance of multi-targeted therapies that can be effective against this multifactorial nature. In this context, coumarin-based hybrid molecules have been developed. Coumarin derivatives for targeting specific mutations associated with gynecological cancers include compounds such as 4-hydroxycoumarin-platinum (IV) complexes, *o*-prenylated coumarin derivatives, and acylhydrazone and 4-chlorocoumarin-modified 1,5-diarylpyrazole benzenesulfonamide derivatives. It is postulated that these derivatives may have potential therapeutic effects by targeting specific mutations in gynecological cancers.

These studies present novel molecular-level approaches to address current challenges in gynecological cancer treatment. In particular, they provide important advances in areas such as overcoming platinum resistance, targeting HPV-associated cancer pathways, and personalizing treatment strategies based on genetic mutations. The findings of these studies may inform the development of clinical applications for more effective treatment of gynecological cancers. The use of coumarin-based hybrid molecules in the treatment of a wide range of conditions is becoming increasingly common. These molecules are able to affect multiple biological pathways, providing an effective treatment across a broad spectrum of conditions. These molecules can inhibit the proliferation of tumor cells and induce apoptosis by overcoming resistance mechanisms. Studies have demonstrated that coumarin derivatives exhibit antiproliferative properties in cancer cells, thereby underscoring the potential of multi-targeted therapies in cancer treatment.

An overview of the described compounds and their potential mechanisms of action is provided in Table 4.

**TABLE 4:** Summary of anticancer effects of coumarin hybrids in gynecologic cancer.

Compound	Cell line	Concentration	Mechanism	References
[PtCl_2_(H-MeOBC) (DMSO)], [Pt_2_Cl_3_ (MeOBC) (DMSO)_2_]	HeLa SK-OV-3	0.5 ± 0.2 mM 10.3 ± 0.3 mM	Cell cycle arrest at G2/M Mitochondrial dysfunction Telomerase inhibition	Qin et al. (2018)
1,2,4-triazole, 4,5-dicyanoimidazole, and purine	HeLa	35.5 ± 13.5 μM	Inactivation of the p53 and pRB tumor suppressors	Benci et al. (2012)
2-[bis(tert-butoxycarbonyl)amino]-5-methylpyridine 1, bromomethyl derivative	HeLa	2.17 ± 0.45 μM to >50 μM	PI3K/Akt pathway inhibition of Akt phosphorylation Apoptosis	Ma and Liu (2017)
2-substituted acetoacetate 2, tert-butylated coumarin 3, pyridinylureas				
3-(coumarin-3-yl)-acrolein derivatives	HeLa	0.195 and 0.78 μM	Apoptosis through the PI3K/AKT-mediated Bcl-2 signaling pathway Inhibiting cell proliferation, migration, and invasion and promoting apoptosis	Chen et al. (2023)
4,7-dihydroxycoumarin-based acryloylcyanohydrazone derivatives	HeLa	6.19 μM	Induces the G2/M phase and inhibits tubulin polymerization	Govindaiah et al. (2019)
5-chloro-n-(2-methoxy-5-(methyl (2-oxo-2H-chromen-4-yl)amino) pentanamide	A2780s A2780T	33.7 ± 12.4 nM	Inducing cell cycle arrest at the G2/M phase, inhibiting cell proliferation, and promoting apoptosis	Cao et al. (2016)
Cinnamoylchromen-2-one	SK-OV-3	14.5 µM	Src kinase inhibition	Chand et al. (2013)
Condensed tricyclic coumarin sulfonate derivatives	OVCAR-4	8.13 µM for 1 h, 5.76 µM for 1i	Stronger antiproliferative activities were observed in aromatic sulfonate derivatives compared to aliphatic and cyclopropyl analogs	El-Gamal and Oh (2014)
Coumarin–nifurtimox (RKS262)	OVCAR-3	∼3 μM	Decrease in mitochondria transmembrane depolarization Pro-apoptotic and pro-survival Bcl-2 family proteins in ovarian cancer cells	Singh et al. (2011)
Coumarin–benzimidazole	OVCAR-8 SK-OV-3	0.07–3.57 µM	Inhibition of PI3K-AKT-mTOR signaling	Liu et al. (2015)
Coumarin–benzimidazolium salts	A2780	100 μM	21.55% ± 5.06%; −66.52% ± 12.43% inhibition	Karataş et al. (2019)
Coumarin–benzylsulfone	HeLa	18.1–32.6 µM	PI3K inhibition	Wang et al. (2019)
Coumarin–chalcone	HeLa C33A	3.59–81.10 µM	Induces G2/M arrest and apoptosis	Sashidhara et al. (2010), Singh et al. (2014)
Coumarin–furoxan	A2780 HeLa SKOV3 OVCA429 OVCA433	20.9–445 nM	Induces G2/M arrest and apoptosis	Liu et al. (2014); Wang et al. (2018); Guo et al. (2018)
	HeLa	10.21 µM	Induces G2/M arrest and apoptosis	Zhang et al. (2018)
Coumarin–maltol hybrids	HeLa	2.47–4.26 μM	Compounds 10j and 10k cleave DNA Methoxy substitution at the C-6 position enhances anticancer activity	Koparde et al. (2018)
Coumarin–palladium (II) complexes	HeLa	5.68 ± 0.69 μM for C1 4.25 ± 0.85 μM for C2	Proapoptotic effects RTK inhibition Pd(II)–coumarin complexes GFR and BCL-2 inhibitors	Avdović et al. (2022)
Coumarin–sulfonamide	HeLa	0.36–40.14 µM	Triggers apoptosis in cancer cells Prevents metastasis Binds positively to COX-2 Selective inhibitor	Lu et al. (2016)
Coumarin–thioxopyrimidine	HeLa	10 µM	Inhibition of p38 MAPK phosphorylation	Batran et al. (2017)
Hybrid series A-I (21.26a,a-d) and series A-II (21.26aʹ-21.26dʹ	A427 SISO	0.01–0.30 µM	Induces apoptosis in A427 and SISO cell lines SAR indicates benzoxazole or benzothiazole ring enhances activity	Makowska et al. (2019)
Iridium (III) coumarin salicylaldehyde Schiff base	HeLa	9.9 ± 0.1 µM to 40.7 ± 12.9 µM	The energy-dependent mechanism for cellular uptake	Liu et al. (2020)
O-prenylated coumarin	HeLa	30 µM	Induces G1/S arrest and apoptosis	Maleki et al. (2020)
Platinum (II) complexes (Pt1–Pt8)	SK-OV-3/DDP	1.01–10.32 μM	Pt1 induces apoptosis in approximately 43.0% of SK-OV-3/DDP tumor cells. Telomerase inhibitor. Mitochondrial dysfunction	Meng et al. (2018)
Shikonin coumarin carboxylic acid	HeLa	3.25 ± 0.35 µM	Reducing mitochondrial membrane potential and disruption of glycolysis promotes apoptosis Induction of apoptosis by downregulation of the expression of HIF-1	Han et al. (2018)
The diethylene glycol-bound isatin–coumarin hybrids	HeLa SK-OV-3	10.28- > 50 µM	Inducing apoptosis, inhibiting cell proliferation, and targeting specific molecular pathways Causing DNA damage and displaying anti-angiogenic effects	Fan et al. (2018)
Thiazol-hydrazono-coumarin	HeLa	0.0091–0.5689 µM	Activation of caspases-9 and -3 inducing cell apoptosis	Abd El-Karim et al. (2019)
Triphenylethylene-coumarin	HeLa	10 μM	Interaction with DNA; disrupting processes vital to cancer cell survival Inhibition of cancer cell proliferation and DNA binding	Tan et al. (2014)

## 5 Pharmacokinetics of coumarins

After oral administration, coumarin is completely absorbed through the GI channel and extensively metabolized in the liver during the first pass. Only 2%–6% of the drug reaches the systemic circulation unchanged (Ballazhi et al., 2023). The liver enzyme CYP2A catalyzes the conversion of coumarins to 7-hydroxycoumarins (Vassallo et al., 2004). The hepatic microsomes initially metabolize it through a specific cytochrome P-450 monooxygenase (CYP2A6) system (Küpeli Akkol et al., 2020; Qi et al., 2022), resulting in hydroxylation before phase II conjugation. The primary phase II metabolite is a glucuronide conjugate. Coumarin can be metabolized by hydroxylation at six possible positions (C-3, 4, 5, 6, 7, and 8). However, hydroxylation most commonly occurs at positions 3 and 7, resulting in 7-hydroxycoumarins (7-OHCs) and 3-hydroxycoumarins (3-OHCs), respectively. In humans, coumarin is metabolized to 7-OHC. During phase II conjugation, 7-hydroxycoumarin is rapidly converted to glucuronide, forming 7-OHCG in the intestine and other tissues. Coumarin and its metabolites are rapidly excreted in urine. Egan et al. discovered that, on average, 63% of a total dose of 200 mg of coumarin was recovered as 7-OHCs in the urine of volunteers over 24 h, with the majority being excreted within the first 24 h. The remaining amount was excreted in the blood. During the first 10 h, most of this was eliminated (Egan et al., 1990).

With percentages of 0.22% and 0.03%, both coumarin and 7-hydroxycoumarin have low aqueous solubility, respectively. This suggests that their *in vivo* bioavailability is also low. An aqueous solubility of 0.3% is considered to be the critical value at which the absorption rate of the compound is limited by its dissolution. The poor bioavailability and half-life of 7-hydroxycoumarin make its *in vivo* relevance questionable. Rapid absorption in aqueous solutions is facilitated by the high partition coefficients of both compounds. Coumarin can easily cross lipid bilayers by passive diffusion due to its non-polar nature (Cooke, 1999).

In a study conducted by the authors, it was shown that the administration of repeated doses of a slow-release coumarin preparation resulted in increased plasma levels of the drug. This was further supported by detecting 7-OHC in urine. Liposomes were used to encapsulate coumarin, with the aim of achieving slow release and metabolism of the compound. Due to coumarin’s low solubility in aqueous solutions and low encapsulation, this was not very successful. Because of its rapid metabolism to 7-OHC, coumarin is often considered a prodrug. As coumarin has a short half-life and low bioavailability, this hypothesis has been partially supported. High-protein lymphedema (HPLO) studies have shown that 7-OHC is effective in many forms of HPLO up to 2 weeks post-dose. It was originally thought that cells could store coumarin, but pharmacokinetic studies have shown that this is not the case (Egan et al., 1990).

As patients taking anticoagulants have comorbidities that require drug therapy, the issue of drug interactions with anticoagulants is extremely important. In particular, due to their anticoagulant properties that can lead to hemorrhaging, drug interactions with coumarins have emerged as a potentially serious clinical problem (MacLeod and Sellers, 1976).

The interaction between vitamin K and coumarin is the most significant and common drug interaction with oral anticoagulants. Oral anticoagulants are believed to compete with vitamin K for a receptor within the liver cell. This receptor is normally required as a cofactor for the synthesis of coagulation factors II, VII, IX, and X. As a result, the synthesis of the prothrombin complex is interfered with. The significance of the coumarin effect on the intestinal production and bioavailability of vitamin K is crucial. Coumarin anticoagulants are usually absorbed through passive diffusion in the upper gastrointestinal tract. The highest plasma concentrations are typically observed between 3 and 9 hours after dosing (Weser and Sellers, 1971). Certain drugs, including allopurinol, cholestyramine, griseofulvin, and heptabarbitone, have been suggested to decrease the completeness of coumarin absorption in the upper gastrointestinal tract. Additionally, antacids such as aluminum hydroxide are expected to increase alkalinity, leading to increased ionization and decreased absorption of the oral anticoagulant due to the weakly acidic nature of coumarin anticoagulants. Cereals (phytates) and mineral oil have also been found to reduce warfarin absorption (MacLeod and Sellers, 1976).

Coumarin anticoagulants are highly protein-bound in the circulation (90%–99%), and free anticoagulant concentration interacts with hepatocellular receptors, affecting synthesis of clotting factors II, VII, IX, and X. Consequently, a significant increase in coumarin-induced hypoprothrombinemia is predicted for any drug that increases the serum-free anticoagulant concentration (Deykin, 1982). The summary of pharmacokinetics of coumarin hybrids in gynecologic cancer is summarized in Table 5.

**TABLE 5:** Summary of pharmacokinetics of coumarin hybrids in gynecologic cancer.

Drug/Formulation	Key Findings	Successes	Failures	Study
Coumarin (oral)	Complete absorption through GI tract, extensive first-pass metabolism, and 2%–6% reaches systemic circulation unchanged	Established absorption and first-pass metabolism data	Low systemic availability	Ballazhi et al. (2023)
Coumarin	Metabolized to 7-hydroxycoumarins by CYP2A	Identified metabolic pathway	N/A	Vassallo et al. (2004)
Coumarin	Metabolized by CYP2A6, leading to hydroxylation before phase II conjugation	Detailed metabolic steps	N/A	Küpeli Akkol et al. (2020); Qi et al. (2022)
Coumarin and 7-hydroxycoumarin	Low aqueous solubility, high partition coefficients, and crosses lipid bilayers by passive diffusion	Identified solubility and diffusion properties	Poor bioavailability and half-life	Cooke (1999)
Coumarin anticoagulants	Significant drug interactions, particularly with vitamin K	Highlighted clinical significance of interactions	Potential for serious hemorrhaging	MacLeod and Sellers (1976)
Coumarin anticoagulants	Highest plasma concentrations observed 3–9 h post-dosing and interaction with vitamin K	Established absorption timeline	Interference with vitamin K metabolism	Weser and Sellers (1971)
Coumarin anticoagulants	Highly protein-bound, free anticoagulant concentration affects clotting factor synthesis	Provided insight into protein binding and clotting factors	Increased risk of hypoprothrombinemia	Deykin (1982)

## 6 Toxicity of coumarins

Coumarin has been shown to cause liver damage at high doses. Synthetic coumarin as a food additive was banned in the United States in 1954 (Hazleton et al., 1956). Prolonged exposure to elevated levels of coumarin in *in vivo* experiments has been linked to liver toxicity, which can lead to liver damage or failure over time (Lake, 1999). Regulatory bodies such as the European Food Safety Authority (EFSA) have set maximum levels for coumarin in certain foods and beverages. The Scientific Committee on Food of the European Commission recommended a maximum level of 0.5 mg/kg of coumarin in food. In 2004, the European Food Safety Authority (EFSA) Panel revised these recommendations (EFSA, 2004), which were originally made by the SCF. The revision was based on new evidence of a non-genotoxic mode of action. As a result of this revision, a tolerable daily intake (TDI) could be derived for the first time (Abraham et al., 2010).

Effects observed *in vivo* include carcinogenic and hepatotoxic properties. Hepatotoxicity has been observed in many mammalian species, in addition to rodents. Long-term studies in rodents have shown tumorigenicity, including adenomas and cancers of the liver and bile duct, adenomas and cancers of the kidney in rats, and adenomas and cancers of the lung and liver in mice. Carcinomas were only observed at doses greater than 100 mg bw per day (Edwards et al., 2000; Api, 2001).

Numerous *in vivo* and *in vitro* studies have been conducted on laboratory animals to investigate the mechanism of coumarin-related hepatotoxicity and clarify coumarin metabolism. The main pathways of coumarin metabolism include 7-hydroxylation, which leads to detoxification and is prevalent in primates, and the metabolism of the lactone ring to form the coumarin-3,4-epoxide intermediate (Pearce et al., 1992; Lake, 1999; EFSA, 2004).

Humans metabolize coumarins in various ways, including coumarin 7-hydroxylation, which is an important detoxification pathway. However, it is important to note that the formation of some metabolites can be toxic. According to Vassallo et al., the CYP2A enzyme in the liver catalyzes the conversion of coumarins to 7-hydroxycoumarins (Ashraf et al., 2017). Additionally, linear furanocoumarins can be phototoxic. However, it has been shown that umbelliferone is non-toxic, indicating that various types of coumarins may have distinct biological effects (Garg et al., 2020).

Coumarins have been shown to lower the risk of ovarian and cervical cancer. However, high doses of coumarin can affect blood clotting and cause liver toxicity. Therefore, coumarin consumption is regulated, and maximum levels are set for certain foods. When consuming foods and products containing coumarin, it is important to take care and minimize health risks. Regulatory bodies, such as the European Food Safety Authority (EFSA), have established maximum levels of coumarin in certain foods and beverages to mitigate potential health risks associated with its consumption (Abraham et al., 2010).

Coumarins may be hepatotoxic due to their structural similarity to aflatoxins. However, substituting coumarins can eliminate their toxic effects and, in some cases, even have a beneficial effect. The difference in hepatotoxicity between rats and humans suggests that these compounds may have varying effects on different species. Although certain coumarins, such as psoralen, have potential toxicity, they may have therapeutic effects that could be beneficial in treating conditions like psoriasis (Asif, 2015). Coumarins have anticoagulant effects that may increase the risk of bleeding, particularly when taken in large doses or combination with other blood-thinning medicines. Although high doses of coumarins have been shown to cause liver damage, they are also beneficial in reducing the risk of cancer as well as brain and cardiovascular diseases.

## 7 Conclusion

Due to inadequate diagnosis of cancer, limited prevention options, and lack of effective treatment, cancer-related mortality rates are alarming today. Coumarins, both natural and synthetic, are of great industrial interest due to their wide distribution and their biological and pharmacological properties due to their distinctive structural scales. This compilation discusses coumarin, which is a powerful bioactive molecule in gynecological cancers.

Scientific studies have shown that coumarins have antitumor activity, depending on their effects on cell growth/differentiation and immune regulation. The toxic effects of coumarins, which make up a significant proportion of our daily diet, are concentration-dependent, and it is difficult to achieve a toxic dose with a normal diet. Although there are restrictions on the use of most natural coumarins due to their hepatotoxic effects, relatively safe analogs with greater potential have now been obtained through molecular modifications. Significant positive results have been obtained in screening for anticancer activity by adding substituents at different positions of the coumarin nucleus. The 3rd and 4th positions of the coumarins are considered to be more pronounced in terms of biological activity. The non-redox properties of the dihydroxy group associated with simple play are inappropriate *in vivo*, and a modification of the pyrone cycle due to its side effects eliminates this condition. It is highly likely to become an anticancer agent by hybridization with different species. The potential for using compounds in this class as drugs to treat gynecological cancers is great. More detailed preclinical studies are needed to assess the efficacy, safety, and pharmacokinetic properties of coumarin in the treatment of gynecological cancers.
